# Multifunctional Hydrogels in Sustainable Agriculture: Structure Design, Application and Future Challenges

**DOI:** 10.3390/gels12090763

**Published:** 2026-08-26

**Authors:** Hanyu Huang, Luohui Wang, Xiaobo Xue, Man Yin, Liyun Wang, Youming Dong, Fei Xiao, Xiangmeng Chen, Cheng Li, Xin Guo, Xian Wang, Lin Zhang

**Affiliations:** 1 College of Horticulture, Henan Agricultural University, Zhengzhou 450026, China; 2 College of Forestry, Henan Agricultural University, Zhengzhou 450026, China; 3 College of Materials Science and Engineering, Nanjing Forestry University, Nanjing 210037, China; youming.dong@njfu.edu.cn; 4 Hunan Academy of Forestry, Changsha 410018, China; 5 College of Pharmacy, Changsha Medical University, Changsha 410219, China

**Keywords:** hydrogel, agricultural water retention, water regulation mechanism, sustainable agriculture, crop resilience

## Abstract

Confronted with severe global challenges, including water scarcity, excessive use of chemical fertilizers and pesticides, and heavy metal contamination in soils, conventional agricultural technologies exhibit marked limitations in integrated water–fertilizer management and non-point source pollution control. Leveraging their excellent water retention capacity, intelligent sustained-release properties, and environmental responsiveness, hydrogels offer innovative solutions to advance sustainable agricultural development. This review comprehensively outlines the fundamental types, crosslinking mechanisms, and key functional properties of hydrogels, with a focused discussion on their agricultural deployment as high-efficiency soil conditioners, fertilizer vectors, and pesticide carriers; it deciphers the microscopic water-holding mechanisms under the tristate water model, delineates the divergent water-uptake and retention behaviors between ionic and non-ionic hydrogels, and clarifies the cyclic water-holding and release mechanisms of hydrogels during soil amelioration. Thise paper further synthesizes hydrogel-enabled environmental remediation applications, in which heavy metals and pesticide residues in soils and aquatic systems are removed via functional-group coordination adsorption or photocatalytic degradation; concurrently, hydrogels have been shown to activate plant systemic immunity through calcium-signaling pathways, thereby inducing broad-spectrum antiviral defense responses. Moreover, hydrogels can be integrated into precision agriculture frameworks to enable real-time monitoring of crop physiological status and to support targeted irrigation and fertilization management. This work also evaluates the role of hydrogels in promoting seed germination, root system development, crop metabolic regulation, and stress resilience, while introducing tailored application strategies across distinct plant growth stages. Their documented economic advantages include water conservation, enhanced crop yields, reduced dependence on synthetic fertilizers, and lower labor costs. Nevertheless, the large-scale implementation of hydrogels continues to face multifaceted challenges—particularly poor degradability and latent ecological risks, as conventional polyacrylamide (PAM)-based gels resist soil mineralization and retain potentially neurotoxic monomers, leaving a critical gap in multi-annual field data concerning their non-target interference with native soil aggregate evolution, pore distribution, and rhizospheric carbon–nitrogen footprints. Mechanistically, many hydrogels with tensile strengths below 1 MPa are highly susceptible to three-dimensional network collapse under high-salinity osmotic shock and tillage mechanical stress, exhibiting a precipitous drop in water retention after more than three wet–dry cycles due to deficient long-term structural stability. Compounding these technical gaps are elevated production costs and low farmer adoption, driven by the absence of texture-specific performance thresholds—such as an available water increment ≥ 40% for sandy soils—and the lack of established life-cycle cost models and farmer incentive mechanisms for bio-based hydrogels. Moving forward, hydrogel technology should pivot toward materials innovation and cost-reduction engineering to broaden its applicability, employ ≥3-year, multi-habitat regional trials to delineate ecological benefit–risk boundaries, and ultimately position hydrogels as pivotal enablers of sustainable, green agricultural paradigms.

## 1. Introduction

Currently, global agriculture is facing severe challenges. The continuous growth of the global population, along with climate change and extreme weather conditions, is intensifying the resource and environmental pressures on agricultural production systems. Problems such as water scarcity, insufficient irrigation water, pollution from excessive fertilizer use, heavy metal contamination, and pesticide abuse severely restrict the sustainable development of agriculture and directly threaten crop yields and food security [1]. Traditional solutions often fail to balance the efficiency and accuracy of water and fertilizer management, as well as the cost and ecological safety of pollution control, presenting significant technical bottlenecks. Therefore, in this context, it is particularly urgent to develop new functional materials to overcome current agricultural technical difficulties and to create efficient, environmentally friendly materials and technologies for agricultural water resource management [2]. In recent years, with the cross-fusion of materials science and agricultural engineering, hydrogels, as functional materials formed by cross-linking hydrophilic polymers into a three-dimensional network structure, have achieved significant development [3]. Hydrogels have evolved from simple inorganic salt gels to materials with environmental response characteristics, gradually overcoming the limitations of traditional mechanical properties. They have now developed into key materials for promoting agricultural green transformation, featuring multifunctional characteristics, excellent biocompatibility and degradability, as well as more precise intelligent and dynamic response capabilities [1].

Hydrogels demonstrate significant potential in the agricultural field. They can dynamically absorb and release water, pesticides, and fertilizers through diffusion and penetration regulation mechanisms, as well as in response to soil moisture gradients and temperature changes, significantly improving the efficiency of water and fertilizer utilization [2]. With breakthroughs in dynamic covalent bond reconstruction and structure optimization technologies in recent years, the application of hydrogels in agriculture has continuously expanded [1]. Hydrogels can precisely respond to external stimuli such as temperature, pH, and light, enabling the targeted delivery of plant hormones, fertilizers, and microbial preparations [4]. Meanwhile, functional groups such as carboxyl (-COOH), sulfhydryl (-SH), and amino (-NH_2_) contained within the hydrogel’s network structure can specifically recognize and efficiently adsorb heavy metal ions, including cadmium (Cd^2+^), lead (Pb^2+^), and arsenic (As^3+^) through coordination bonds, electrostatic attraction, and chelation, thereby removing these heavy metal ions from the environment and effectively reducing their harm to organisms [5].

Although hydrogels have many applications in the agricultural field and show significant advantages over traditional methods, they still face many challenges [6]. For example, the cost of producing hydrogels is relatively high, which may be difficult for ordinary farmers to bear. Additionally, the production steps are complex and difficult for most ordinary farmers to master. Furthermore, the synthesis of some hydrogels relies on industrial products such as petrochemicals, which may be difficult to degrade in nature and cause environmental pollution [7,8]. Hydrogels may pose potential risks to soil health and ecosystem sustainability during long-term use. This article systematically explores the types, properties, preparation methods, and agricultural applications of hydrogels, as well as their effects and mechanisms in improving soil water retention, nutrient transport, and crop yields [6]. Unlike previous reviews that mainly focused on the single functions of hydrogels, this review systematically integrates the multiple roles of hydrogels in sustainable agriculture—covering from the microscopic “three-state water” mechanism for soil moisture regulation, the fundamental differences in water absorption and retention behaviors between ionic and non-ionic hydrogels, to their functions as intelligent carriers for fertilizers/pesticides, heavy metal adsorbents, photocatalytic degradation platforms, and the activation of plant systemic immunity through calcium signaling pathways, and further constructs a comprehensive framework that encompasses the entire process from material design and soil improvement to crop response, environmental restoration, and precision agriculture. Meanwhile, for the first time, this paper explicitly proposes differentiated application strategies for different soil textures and different growth stages. Based on the analysis of specific technical bottlenecks such as the poor degradability of polyacrylamide-based hydrogels and the network collapse under high-salt environments, a future R&D path is put forward, which includes quantitative performance thresholds such as a water retention rate of hydrogels ≥ 60% after multiple field dry–wet cycles and an in situ degradation rate ≥ 70% within 80 days, as well as multi-regional and multi-year field verifications. Thus, the discussion is advanced from the proof-of-concept stage to the levels of engineering and ecological safety assessment.

## 2. Types and Properties of Hydrogels

### 2.1. Classification of Hydrogels Based on Synthesis Methods

Hydrogels used in agriculture can be classified into three categories based on their synthesis methods: traditional synthetic hydrogels, biodegradable hydrogels, and hybrid hydrogels. These are explained below:

Traditional hydrogels mainly consist of acrylamide, acrylic acid, or acrylate salts crosslinked chemically [6]. Traditional hydrogels are widely used in agriculture due to their relatively low cost and are well received by farmers. Their advantages include high water absorption capacity, good mechanical strength, and the ability to control structure and performance effectively through synthesis processes. They are often used as soil conditioners to improve soil moisture retention in arid and semi-arid regions. They provide effective control over structure and performance through adjustable synthesis processes, enabling customization to specific agricultural needs. It significantly improves water-use efficiency and reduce the frequency of irrigation. However, some traditional synthetic hydrogels are difficult for soil microorganisms to degrade, and their large-scale use may leave harmful residues in the soil, posing a risk of secondary pollution.

Biodegradable hydrogels are mainly derived from animals, plants, or microorganisms, such as gelatin, chitosan, sodium alginate, cellulose and lignin extracted from agricultural waste. They are usually formed into physical gels or chemical gels through physical crosslinking (e.g., hydrogen bonds and electrostatic interactions) or mild chemical crosslinking. These gels have good biodegradability. They are environmentally friendly. Their degradation products are generally harmless to the soil and may even serve as nutrients. Additionally, they have a wide range of raw material sources and are renewable [5]. For example, a research team at Jiangnan University developed a fully biodegradable gelatin hydrogel seed-coating technology. This technology can promote plant growth, support sustainable crop development and food security, reduce labor and pesticide use, and be environmentally friendly and efficient. It can also maintain a balance between high crop yields and low environmental pollution. Biodegradable hydrogels not only enable the development of sustainable, environmentally friendly soil conditioners but also facilitate biodegradation by soil microorganisms, thereby reducing the environmental risks associated with traditional synthetic hydrogels [9]. However, some natural polymer hydrogels may exhibit relatively poor stability and mechanical strength, which are inferior to those of synthetic hydrogels. By combining natural and synthetic materials, three-dimensional hydrogels with well-defined structures can be prepared that exhibit superior performance compared with natural hydrogels in modern agriculture.

Hybrid hydrogels are prepared by combining natural and synthetic polymers or by doping the hydrogel matrix with inorganic nanoparticles, such as nano-TiO_2_ [10,11]. These hydrogels aim to overcome the limitations of single materials and achieve synergistic enhancement effects. For example, these hydrogels are designed to overcome the limitations of a single material and achieve a synergistic enhancement effect. For instance, they can achieve extremely high water absorption rates, excellent mechanical strength, or intelligent responses to environmental factors such as temperature, light, moisture, and pH value, thereby enabling more precise control of water or nutrient release [12]. In addition, it has great potential to alleviate metal-ion adsorption and cadmium toxicity and to make a significant contribution to diverse application-oriented fields [13]. These hydrogels can be used extensively in different agricultural fields, the cosmetic industry, and biomedical applications due to their biocompatible, biodegradable, and biosorbent nature [14,15].

### 2.2. Classification of Hydrogels Based on Crosslinking Mechanisms

The crosslinking mechanism of hydrogels plays a crucial role in their structural integrity, responsiveness, and swelling behavior [16]. The pore structure in hydrogels refers to the spatial voids or gaps within the three-dimensional polymer network. These pores form during the crosslinking process and play a critical role in determining the hydrogel’s water absorption, retention, and release. Smaller pores primarily influence water binding and slow diffusion, while larger pores facilitate rapid water transport and nutrient release. Therefore, controlling pore structure is essential to tailoring hydrogel performance to specific agricultural applications, such as soil moisture retention or controlled fertilizer delivery. Based on the nature of the forces that form crosslinking points in the three-dimensional network structure of hydrogels, hydrogels can be classified as physical, chemical, or multi-crosslinked [17].

**Figure 1:**
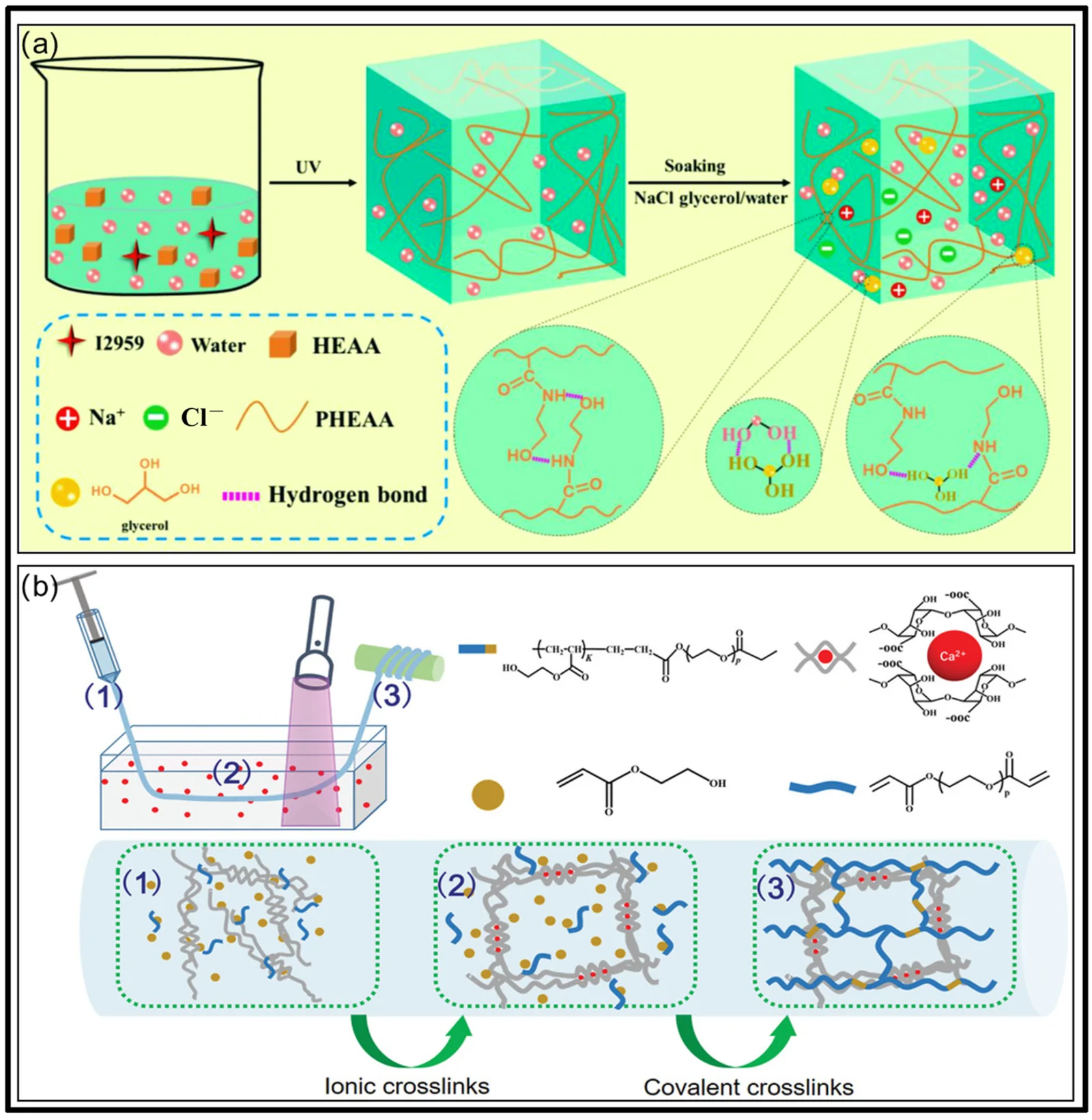
(**a**) Preparation of PHEAA–Gl–NaCl physical hydrogel. Reproduced with permission from reference [20]. (**b**) Molecular design of the mixed cross-linked polymer network in organic hydrogel fibers, along with a schematic illustration of the wet-spinning process and molecular evolution of the hydrogel fiber. (1), (2), and (3) show structural evolution at different stages during spinning, including: (1) spinning solution, (2) ionically cross-linked fiber, and (3) mixed cross-linked fiber. Reproduced with permission from reference [21].

Physically crosslinked hydrogels form three-dimensional network structures through non-covalent interactions such as ionic bonds, hydrogen bonds, hydrophobic interactions, van der Waals forces, or molecular chain entanglements [18,19]. Such crosslinking processes generally do not require chemical crosslinking agents, occur under mild conditions, and are reversible, which endows hydrogels with stimulus responsiveness and self-healing potential. However, physically crosslinked hydrogels typically exhibit relatively low mechanical strength, and their structural stability is susceptible to environmental factors. A typical example is sodium alginate-calcium ion crosslinked hydrogels: sodium alginate is a natural polysaccharide, and the guluronic acid units along its molecular chains can form ionic coordination bonds with divalent cations, thereby forming a crosslinked network. In Figure 1a, the PHEAA–Gl–NaCl physical hydrogel is composed of PHEAA (or PVP) as the polymer framework. It is dissolved in a mixture of water, glycerol, and a small amount of NaCl, heated to dissolve, and then cooled to form. There are no covalent cross-linking agents. The three-dimensional network is maintained solely by non-covalent physical nodes and exhibits thermoreversible and self-repairing properties. It is a typical fully physical cross-linked hydrogel [18].

Chemically crosslinked hydrogels form permanent three-dimensional networks by covalently bonding polymer chains [22]. Such hydrogels generally require crosslinking agents (e.g., glutaraldehyde, genipin), initiators (thermal or photoinitiation), or high-energy radiation to initiate polymerization or crosslinking reactions [23,24]. Their network structures are stable, and their mechanical properties are excellent; however, the crosslinking process may introduce biologically toxic substances, and the crosslinked structure is irreversible [24]. For instance, when oxidized hyaluronic acid is mixed with succinyl chitosan, the two components form hydrogels via in situ crosslinking through a Schiff base reaction. In Figure 1b, using acrylamide (AM) as the monomer and N,N’-methylene bisacrylamide (MBA) as the covalent crosslinking agent, the mixture of water and DMSO was dissolved, and then heated/UV-initiated free radical polymerization was carried out to form a polyacrylamide (PAM) covalent three-dimensional network. Within the hydrogel, MBA forms irreversible covalent bonds at crosslinking points, and there are no physical nodes in the second network. This is a typical chemical hydrogel [19].

Multiple crosslinked hydrogels construct synergistic networks by simultaneously utilizing physical interactions and covalent chemical bonds, thereby integrating the reversibility of physical crosslinking with the stability of chemical crosslinking [25]. Common strategies include constructing double networks or introducing nanocomposites. Such hydrogels can exhibit multiple functional properties, including high strength, high toughness, and self-healing [26]. A representative example is poly(vinyl alcohol) (PVA)/sodium alginate (SA) double-crosslinked hydrogels: the first network is formed via ionic crosslinking between Ca^2+^ and the carboxylate groups of SA, and the second network is formed via physical crosslinking of PVA chains through repeated freeze–thaw cycles, resulting in microcrystalline regions. This significantly improves both the compression modulus and fatigue resistance of the hydrogel. Conversely, the crosslinking, whether physical, chemical, or multi-crosslinking, directly influences the network parameters of hydrogels, including pore size, crosslink density, and the average molecular weight of polymer chains between crosslinking points (Figure 1).

### 2.3. Types of Water in Hydrogels

The different states of water molecules inside hydrogels play a critical role in the properties of hydrogels themselves. Bound water, intermediate water, and free water interact with polymer networks through different modes, and together determine the mechanical properties, environmental stability, microbial inhibiting properties, and functional performance of hydrogels [27]. The “three-state” model of water in hydrogels has undergone long-term development, which categorizes water in hydrogels into “bound state”, “intermediate state”, and “free state” [28,29].

Bound water strongly combines with the hydrophilic groups of polymer chains through hydrogen bonds, and its movement is severely restricted. It does not freeze even at temperatures as low as −30 °C [30,31]. The bound water can enhance the toughness of the hydrogel and prevent the formation of ice crystals at low temperatures, which can damage the network. An appropriate proportion of bound water can enable the hydrogel to maintain elasticity, transparency, and normal function in extremely cold environments [32]. Intermediate water exhibits an interaction strength between that of bound water and free water, with partial restriction, and its phase transition temperature is below 0 °C [33]. Intermediate water represents excellent performance in energy conversion applications. For example, in solar-driven water evaporation and photocatalysis, increasing the intermediate water content in hydrogel can significantly reduce the energy barrier required for water evaporation or chemical reactions, thereby greatly improving efficiency [27]. Free water is loosely stored in the pores of the hydrogel network, is almost not bound by the polymer, and can flow freely. Typically, the freezing point of free water is close to 0 °C [34]. When the hydrogel dries, free water is the first to be released and plays a key role in the expansion and dissipation dynamics of the material [35]. Free water affects solute diffusion and drug release, influences the flexibility of the hydrogel, and has a certain lubricating effect. However, an excessive proportion of free water will dilute the network, leading to a decrease in mechanical strength. For hydrogels that require adhesion, an excessive proportion of free water can weaken adhesion due to swelling and surface water films. By increasing the proportion of bound water through methods such as hydrophobic modification of the polymer, the proportion of free water can be reduced, enabling the hydrogel to achieve rapid and strong adhesion even in humid environments [36].

### 2.4. Properties of Hydrogels

Swelling capacity: Hydrogels can significantly expand upon contact with water, absorbing and retaining several times or even dozens of times their own weight of water. The three-dimensional cross-linked network inside the hydrogel prevents polymer chains from diffusing and dissolving indefinitely in water [37]. The swelling capacity of hydrogels depends on the hydrophilic groups of the polymer chains and the cross-linking density [38]. Generally, the more cross-linking points there are, the denser the network structure and the smaller the pore space, which may lead to a decrease in the swelling degree after water absorption [39].

Mechanical strength: Although hydrogels contain a large amount of water and are soft and elastic, their mechanical strength is often not high, and they are prone to rupture [40,41]. The mechanical strength of hydrogels is affected by their cross-linking density. Hydrogels with a high cross-linking density often exhibit higher mechanical stability and increased durability in various environmental conditions [42]. The internal structure of hydrogels can also help enhance their mechanical strength. For example, a dual-network hydrogel, which interweaves a rigid and brittle network with a soft and tough network, achieves a combination of high strength and high toughness [43,44].

Biodegradability: Hydrogels decompose over time and do not leave harmful residues in the environment [45]. This property is both a key characteristic of hydrogels and a factor that provides possibilities for their use in sustainable agriculture. In contrast, some hydrogels are synthesized using e certain biologically toxic cross-linking agents, and incomplete removal of their residues may affect biocompatibility and raise concerns about the long-term environmental impact of hydrogels [46,47].

Environmental responsiveness: Hydrogels can be designed to respond to specific environmental triggering factors and sense minute changes in the external environment, such as temperature, pH, or ionic strength [48,49]. For example, certain thermosensitive hydrogels will expand or contract at specific temperatures [50]. pH-sensitive hydrogels expand or contract according to the pH of the soil, making them suitable for targeted delivery of agricultural chemicals [51].

## 3. Applications of Hydrogels in Sustainable Agriculture

With the continuous development of industrialization, agricultural productivity has significantly increased over the past few decades [52]. However, at the same time, resource and environmental constraints have become increasingly severe. Water shortages and low water utilization rates, heavy metal pollution, excessive use of fertilizers and pesticides, frequent extreme weather events, reduced and deteriorating farmland quantity, and water waste caused by irrational irrigation have all become key factors threatening the sustainability of agriculture [53,54].

### 3.1. Application of Hydrogels in Agricultural Water Storage

**Figure 2:**
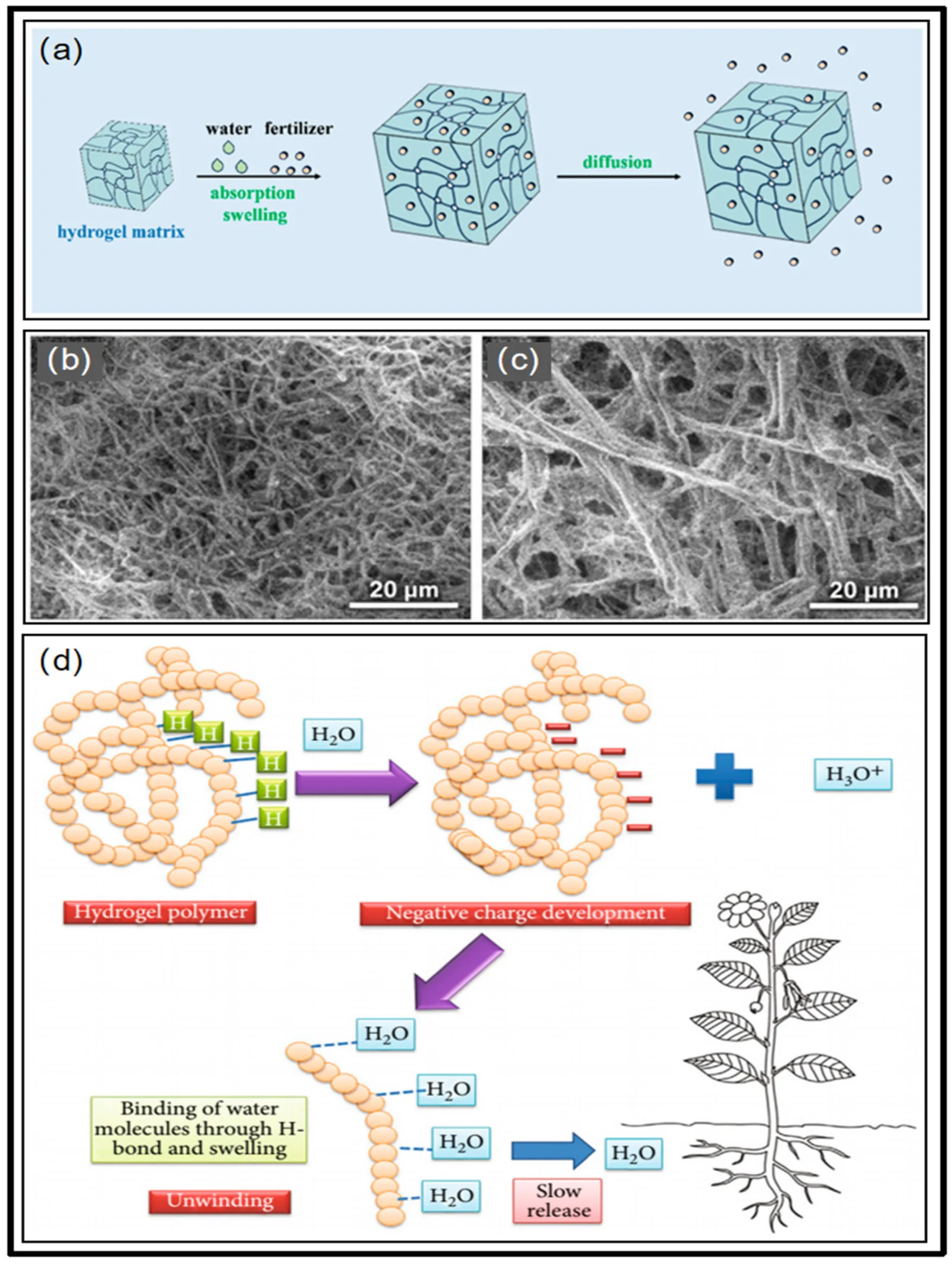
(**a**) Water and nutrient retention and release of hydrogel. Reprinted with permission from reference [7]. (**b**,**c**) Multiscale SEM micrographs of freeze-dried date palm cellulose–alginate composite hydrogel. (**b**) Low-magnification view of the characteristic rough, irregular surface morphology of the composite. (**c**) Higher-magnification cross-sectional views reveal a heterogeneous and interconnected porous network. Reproduced with permission from reference [55]. (**d**) Mechanism of action of hydrogel upon soil-based application. Reproduced with permission from reference [36].

Hydrogels are composed of three-dimensional network structures formed by cross-linked polymer chains. Their water storage effect depends on the hydrophilic groups they contain and the degree of cross-linking within their structure. The hydrogels are rich in hydrophilic groups such as hydroxyl (-OH), carboxyl (-carboxyl), and amino (-amino) groups. Combined with the three-dimensional porous network structure (Figure 2), they provide storage space for water [55]. Figure 2 not only shows the three-dimensional porous network structure of hydrogels, highlighting the absorption and retention of water by the polymer chains, but also demonstrates the gradual release of water as the surrounding soil dries, showcasing the function of hydrogels as a water source for plants. The hydrophilic groups in the polymer chains of the hydrogel are the main reason for the water absorption property of the hydrogel. When the hydrogel comes into contact with water, water molecules are dynamically captured and incorporated into the hydrogel system through intermolecular interactions such as capillary forces, hydrogen bonds, and van der Waals forces of hydrophilic groups. During this process, hydrogen atoms react and are released in the form of positive ions, leaving multiple negative ions along the entire length of the polymer chain. These negative charges repel each other and force the polymer chain to unwind and open up (Figure 2d), and attract water molecules and bind them with hydrogen bonding [56,57]. Hydrogels also have high application value in areas prone to drought or with irregular precipitation due to their ability to absorb, store, and slowly release water [58]. During water scarcity, hydrogels can absorb and store water during irrigation or rainfall and gradually release it when the environment becomes dry, thereby ensuring stable soil moisture, reducing the impact of drought on agriculture, decreasing irrigation frequency, and securing water supply to plants even under difficult conditions, thus achieving more stable crop improvement, plant performance, accelerated yield, and sustainable agriculture [59].

Although the effectiveness of hydrogels in maintaining and enhancing soil water retention has been demonstrated in various soil types, for instance, carboxymethyl cellulose (CMC) hydrogels reinforced with silica, kaolin, and bentonite particles can enhance the water retention capacity in sustainable agriculture [60]. However, the effectiveness of hydrogels in enhancing soil moisture is indeed closely related to soil type [61]. For example, hydrogels show significant effects in soils with poor water retention capacity, such as sandy soils, whereas their effects are relatively limited in heavy clay soils, which already have strong water retention capacity [62]. For example, in sandy soils, the pores are large and drainage is extremely fast, resulting in poor water and fertilizer retention capabilities. Water infiltration is rapid, and the retention rate is low. Additionally, hydrogels can significantly enhance water retention capacity and reduce water loss through deep seepage [63]. When the soil dries, the hydrogel releases the water it has absorbed into the soil, supplying water to plant roots and helping plants resist drought [56]. The soil-water interaction triggered by the expansion of the aerogel helps stabilize the soil structure (Figure 2d). In loamy soils, the proportions of sand, silt, and clay are balanced, the soil structure is reasonable, and the water retention is moderate. The addition of hydrogels can optimize the pore structure, synergize with the soil’s inherent good water retention capacity, and further extend the water availability [64]. Hydrogels can increase the proportion of soil aggregates, improve soil structure, soil physicochemical properties, porosity, color, moisture content, and water–hydrothermal conditions. It is important to apply them in appropriate amounts, as excessive use may affect air permeability, consistency, organic matter, and temperature [65]. In heavy clay soils, hydrogels absorb excess water and release it as needed, helping to prevent waterlogging and maintain the soil’s optimal moisture level. However, the humidifying effect of hydrogels in heavy clay soils is relatively limited [66].

Despite extensive literature demonstrating the water-retention effects of hydrogels across different soil types, current studies still face several key limitations warranting critical scrutiny. First, most studies report only short-term experimental results under a single soil type, lacking systematic comparisons across soil types. For instance, the efficient water retention of hydrogels in sandy soils often comes at the expense of high water absorption capacity, yet their durability under repeated wetting–drying cycles and the actual extent of deep percolation reduction have not been uniformly quantified. Second, studies focusing on clay soils tend to merely emphasize the limited effectiveness of hydrogels but rarely explore the specific thresholds at which excessive hydrogel application reduces soil aeration and impedes organic matter decomposition, leaving recommended application rates without a scientific basis. Furthermore, interactions among soil microbial activity, root exudates, and hydrogel degradation behavior have rarely been considered, even though these factors could substantially alter the actual efficacy of hydrogels over the medium- to long-term. Finally, different studies employ various application methods—such as surface spreading, mixing incorporation, and embedding—and are conducted under disparate test conditions (e.g., greenhouse pot trials versus field experiments), resulting in highly divergent outcomes that are difficult to compare directly, thereby hindering the extraction of generalizable principles. Therefore, future research should prioritize establishing standardized evaluation frameworks and conducting long-term field trials across multiple soil types and climatic regions to address current deficiencies in hydrogel research for agricultural applications.

### 3.2. The Water Absorption and Water Retention Mechanism of Hydrogels

#### 3.2.1. The Microscopic Water Retention Mechanism of the Three-State Water Model of Hydrogels

The water absorption and retention mechanisms of hydrogels are physicochemical processes synergistically governed by the polymer chemical architecture, crosslinking network parameters, and three-dimensional porous morphology. From the perspective of water states, water confined within the hydrogel network can be classified, according to its interaction strength with hydrophilic moieties, into strongly bound water (SBW), weakly bound water (WBW), and near-bulk water (NBW). SBW forms robust hydrogen bonds with highly polar groups—such as amide, hydroxyl, carboxyl, amino, and sulfonic groups—and remains unfrozen below 260–265 K, serving as the core reservoir for water retention; WBW experiences weaker interactions and freezes between 260–265 K and 273 K; NBW behaves similarly to bulk water and crystallizes at approximately 273 K. The driving force for water uptake arises from the coupling of multiple mechanisms. First, hydrophilic groups provide anchoring sites: the polarity sequence of common functional groups follows amide > carboxylic acid > alcohol > ketone/aldehyde > amine > ester > ether, wherein higher polarity corresponds to elevated SBW content. Second, electrostatic repulsion among ionized groups expands the network; when carboxyl groups are deprotonated at high pH or amino groups are protonated at low pH, enhanced intramatrix repulsion between –COO^−^ or –NH_3_^+^ moieties enlarges mesh sizes, further facilitating water ingress. Third, capillarity and osmotic pressure jointly drive imbibition: interconnected porous architectures—classified per IUPAC standards as micropores (<1.4 nm), ultramicropores (1.4–3 nm), mesopores (3–50 nm), and macropores (>50 nm)—accelerate water transport via capillary forces, with macropores and through-pores providing rapid hydraulic pathways [28]. The water retention phase is finely regulated by crosslinking density and mesh size: an increase in crosslinking density reduces mesh size and lowers the average molecular weight between adjacent junction points, thereby suppressing water ingress and diffusivity while concurrently elevating equilibrium water retention. The Flory–Rehner equation describes the equilibrium relationship between crosslinking density (ρ_χ_) and volumetric swelling ratio (Q), representing the thermodynamic balance between osmotic pressure (polymer–solvent mixing free energy) and elastic retractive forces of the network. The drying/dehydration trajectory reveals a hierarchical retention profile: during the first stage, NBW evaporates concomitant with macroscopic network contraction; in the second stage, WBW evaporates and establishes gas–liquid interfaces within pores, where ensuing capillary pressure triggers a glass transition; in the third stage, residual SBW persists via a tetrahedral hydrogen-bonded network manifesting as two-dimensional hydrogen-bonding motifs. Fundamentally, the essence of hydrogel water management lies in a triple synergy—hydrophilic moieties furnishing binding sites via hydrogen bonding and van der Waals forces, ionizable groups expanding the network via electrostatic repulsion, and the crosslinked porous architecture imposing physical confinement and dictating diffusion pathways. By maximizing the SBW fraction and suppressing NBW mobility, hydrogels achieve both high-capacity uptake and prolonged retention. This structural–functional continuum epitomizes why water itself is rightly regarded as the defining “soul” of hydrogels, operating as the central physicochemical bridge linking molecular architecture to macroscopic performance [28].

#### 3.2.2. The Differences in the Water Absorption and Water Retention Mechanisms Between Ionic and Non-Ionic Hydrogels

The water absorption mechanism of hydrogels is also intrinsically modulated by their intrinsic polymer typology. Non-ionic and ionic hydrophilic polymer hydrogels exhibit fundamentally divergent water imbibition behaviors: the former rely predominantly on capillarity and hydrogen bonding between hydrophilic moieties and water molecules to achieve moisture adsorption, whereas the latter (exemplified by alginate-based systems) are driven synergistically by capillarity, electrostatic repulsion, and osmotic phenomena. This mechanistic divergence is quantitatively corroborated in sodium alginate-graft-polyacrylamide (Alg:PAM) systems [67]. Neat polyacrylamide (PAM), functioning solely via capillary entrapment within its interconnected porous architecture and hydrogen-bonding/van der Waals interactions of amide groups (–CONH_2_), achieves a modest equilibrium swelling capacity of merely 9 g/g with a minimal initial swelling rate constant (k_i_) of 0.00967 g/g·h. Upon integrating sodium alginate into the network, however, carboxyl groups (–COOH) undergo deprotonation at the ambient pH of tap water (~6.5) to yield –COO^−^ moieties; the resultant electrostatic repulsion between intra-network carboxylate anions compels three-dimensional network expansion, while the osmotic pressure gradient generated by the differential ion concentration across the network further drives water influx. Consequently, the equilibrium swelling capacities of the 4Alg:1PAM, 1Alg:1PAM, and 1Alg:4PAM formulations reach 65, 41, and 23 g/g, respectively, with their water-uptake kinetics conforming strictly to a pseudo-second-order model (R^2^ > 0.99). During the retention phase, both ionic and non-ionic hydrogels rely on the physical confinement imposed by crosslinked networks, along with van der Waals forces and hydrogen bonding, to immobilize water—for instance, the 4Alg:1PAM formulation retains over 30% of its water content after 5 days at 30 °C and 30% relative humidity. Notably, this system undergoes in-soil degradation via the hydrolytic cleavage of alginate glycosidic linkages, attaining an 85% degradation rate within 5 weeks; the resulting oligo-alginates act concurrently as biostimulants that promote the growth of Eucalyptus seedlings. Collectively, the water management paradigm of Alg:PAM ionic hydrogels can be distilled as follows: –COO^−^-mediated electrostatic repulsion coupled with osmotic pressure furnishes the volumetric driving force for uptake (ionic-exclusive impetus), interconnected macroporosity provides the spatial reservoir, and hydrogen bonding and van der Waals forces deliver the thermodynamic safeguard for retention. This tripartite synergy endows Alg:PAM with markedly superior water-harvesting capabilities over non-ionic PAM analogs while conferring inherent biodegradability and environmental benignity. Ultimately, the system reconciles high-efficiency soil moisture conservation with growth-promoting functionality, establishing a unified paradigm for water-deficit agricultural deployment [67].

#### 3.2.3. The Water Retention-Release Cycle Mechanism of Hydrogels in the Context of Soil Improvement

In the context of agricultural soil amelioration, the mechanism by which hydrogels improve soil water retention is a multilevel, synergistic process governed by interactions among the material, the soil matrix, and the plant. For instance, researchers constructed a biodegradable superabsorbent hydrogel via esterification crosslinking between sodium carboxymethyl cellulose (NaCMC) and hydroxyethyl cellulose (HEC) using citric acid (CA) as the crosslinker; within soil amendment scenarios, this system demonstrates a complete cyclic paradigm of water management: “swelling-driven uptake—network entrapment—rhizosphere-controlled release—degradation.” At the chemical crosslinking level, esterification between the –COOH groups of CA and the –OH groups on NaCMC/HEC chains was evidenced by the newly emerged ester C=O stretching vibration at 1719 cm^−1^ and the relative narrowing of the –OH stretching envelope across 3250–3550 cm^−1^ in FTIR spectra, confirming the successful assembly of a covalently crosslinked network. This architecture, enriched with hydrophilic moieties (–OH, –COOH) along the cellulose backbone, engages in strong polar interactions—hydrogen bonding and van der Waals forces—with water molecules, thereby furnishing both chemical hydration sites and physical entrapment space [68]. Upon encountering irrigation or rainfall, 2% hydrogel-modified soil (HMS) undergoes rapid matrix swelling: soil bulk density declines from 1.16 g/cm^3^ in unmodified soil (UMS) to 1.06 g/cm^3^, and total porosity rises from 53% to 57.1%. After 10 days under open-air conditions, HMS retains approximately 50% more moisture than UMS. The fundamental retention principle lies in the crosslinked network acting as a rhizospheric “micro-reservoir”: physical confinement suppresses deep percolation and surface evaporation, while entrapped water is released to the root–soil interface at a metered rate, ultimately elevating plant water-use efficiency (WUE) by 25.25–45.52%. Concurrently, the cellulose-based hydrogel biodegrades via soil burial; progressive cleavage of ester bonds and network disintegration eliminate the long-term residue risks inherent in petrochemical-derived hydrogels. The water retention mechanism of this system in soil can thus be distilled to a triple synergy: esterification-mediated covalent elasticity providing network confinement, cellulose’s hydrophilic groups supplying hydrogen-bonded hydration sites, and regulated soil porosity governing water transport and release kinetics. It is precisely this “imbibe–store–meter–degrade” cycle that reconciles high-capacity water harvesting, sustained micro-irrigation, and optimized crop water productivity in arid and semi-arid agricultural settings [39,68].

### 3.3. Strategies for Improving the Water Retention Capacity of Hydrogels

Although hydrogels can absorb and retain approximately tens of times their own weight in water or biological fluids without dissolving, dehydration under drought conditions leads to structural collapse, and freezing-triggered phase separation ultimately results in irreversible hydrogel failure [69,70]. To enable hydrogels to cope with diverse challenges, various strategies have been developed to lock in water through different mechanisms, thereby enhancing their environmental adaptability. Table 1 presents several commonly used strategies for enhancing the water retention capacity of hydrogels, along with representative material systems and quantitative indicators of environmental tolerance under each strategy [71].

Encapsulation strategies build physical or chemical barriers on the surface of hydrogels to directly block the evaporation of water molecules. These strategies include hydrophobic and hydrophilic coatings, as well as amphiphilic surface modifications, which achieve stable interfacial bonding through hydrogen or covalent bonds [72]. For example, hydrophobic coatings can reduce water vapor permeability, while the hydrophilic double-layer structure delays the migration of free water through competitive hydrogen bonds. After polydimethylsiloxane (PDMS) encapsulation, the hydrogel retains 80.45% of its weight within 72 h, while the non-encapsulated group retains only 8.94%. Encapsulation significantly improves water retention rate and can also balance mechanical flexibility with biocompatibility [73]. Researchers employed PDMS-based hydrophobic encapsulation to increase the water retention rate of hydrogels from 8.94% to 80.45% over 72 h and to reduce the average dehydration rate by more than 97%. This provides a direct materials science paradigm for the “hydrophilic water storage/hydrophobic evaporation control” bilayer structure of agricultural water-retaining agents. This mechanism further satisfies the functional requirements of water absorption, storage, and slow release when hydrogels are used as agricultural water retainers and soil conditioners. It demonstrates that the hydrophobic shell can reduce water vapor permeability and retard the migration of free water into the soil gas phase, thereby minimizing evaporation and deep percolation, improving root-zone water-use efficiency, and enabling coordinated controlled release of fertilizers and pesticides. However, the specific performance of the hydrogel under field conditions still requires multiscale validation at the soil, pot, and field-trial levels.

Solvent optimization strategies introduce low-volatility solvents, such as polyols and hydrated ionic liquids (HILs), to replace part of the aqueous phase, thereby altering the hydrogen-bond network and the system’s vapor pressure. For example, glycerol or ethylene glycol can form molecular clusters to trap free water, inhibiting ice crystal formation and evaporation. The introduction of glycerol can endow hydrogels with antifreeze properties and enhance their self-healing performance [74], while the ethylene glycol (EG)/water system can maintain the hydrogel’s excellent tensile strength within the temperature range of −60 °C to 60 °C [75]. The application temperature range of its PAA-CNF-HILs ionic gel is from −45 °C to 75 °C [76], while the hydrogel containing LiCl can withstand temperatures as low as −80 °C [77]. The three systems based on ethylene glycol/water, ionic liquid/CNF, and LiCl enable hydrogels to maintain excellent mechanical and water retention/sustained-release properties under extreme temperatures of −60 °C to 60 °C, −45 °C to 75 °C, and −80 °C, respectively. These strategies endow hydrogels with antifreezing, anti-drying, and self-regeneration capabilities under extreme thermal conditions, thereby preventing soil structural damage and water regulation failure caused by low-temperature embrittlement or high-temperature dehydration. Consequently, the stability of root-zone water storage and slow release is maintained, enhancing water and fertilizer use efficiency. Such approaches provide a reliable broad-temperature-range material paradigm for the design of agricultural water-retaining agents and soil conditioners applicable in cold regions and farmlands with significant diurnal or seasonal temperature fluctuations. Nevertheless, their actual water retention efficacy, soil compatibility, and biodegradability at the field scale still require further investigation.

The ionic incorporation strategy utilizes hygroscopic ions such as Li^+^, Ca^2+^, and Cl^−^, or the strong hydration capacity of polyelectrolytes, to convert free water into bound water [77]. Polyvalent ions can form primary and secondary hydration shells, disrupting the hydrogen bond network of water molecules and lowering the freezing point. For example, calcium chloride (CaCl_2_) enables hydrogels to withstand temperatures as low as −58 °C [78]. Polyelectrolytes can achieve ionic hydration through their charged groups, thereby reducing the proportion of evaporable water and enabling them to absorb water from the environment. For instance, the PAM-CS-CaCl_2_ aerogel can absorb atmospheric water as its primary water source and convert it into liquid water, which is stored in the polymer network’s soil-pore channels. As the water absorption increases, the aerogel will gradually expand. When the soil is dry, the hydrogel will release the water it has absorbed, thereby increasing soil moisture content and improving soil structure [79]. This strategy endows the hydrogel with self-regeneration properties. For instance, the LiCl-containing hydrogels can absorb moisture from the environment and exhibit both self-regeneration properties and freeze resistance down to −80 °C [80]. In summary, the ion incorporation strategy effectively enhances the antifreezing and anti-dehydration properties of hydrogels through hydration effects, providing a rationale for developing agricultural water-retaining agents and soil conditioners suitable for cold regions and areas with large diurnal or seasonal temperature fluctuations.

Structural design strategies enable modulation of polymer network topology, providing a directly transferable materials science paradigm for agricultural water-retaining agents and soil conditioners, with preliminary validation already demonstrated in agricultural settings. Examples include single networks (SNs), the introduction of heterogeneous components such as polymeric microcapsules (PMCs) and nanofillers, and the construction of interpenetrating polymer networks (IPNs) and double networks (DNs), which physically restrict water-molecule diffusion [81]. The hollow cavities of PMCs store water via capillary action, while their functional shells achieve dynamic controlled release through bound-water interactions [82]. Nanomaterials such as silica and graphene increase the number of binding sites owing to their high specific surface area. High crosslinking density and microporous structures prolong the diffusion pathways of water molecules, whereas DNs or IPNs enhance network toughness through synergistic effects [83]. For instance, researchers constructed a semi-interpenetrating network (semi-IPN) multifunctional hydrogel (CPAUH) using carboxylated cellulose nanofibers (CNF) as the rigid network and poly(acrylic acid-co-potassium acrylate) (p(AA-co-KAA)) as the flexible network. As a nanoscale rigid network component, CNF increases the number of binding sites for water molecules and nutrient ions due to its high specific surface area and abundant functional groups. The well-designed structure of this hydrogel yields quantifiable agricultural performance: a mechanical strength of 0.169 MPa, a water absorption capacity of 121.65 g/g, and retention of 118 g/g after three absorption–desorption cycles, demonstrating excellent structural stability. After loading urea and humic acid, the hydrogel exhibited sustained release behavior, with cumulative leaching rates of only 66.91% and 92.45%, respectively, over 15 days. When applied at 1.5% to saline–alkali soil, the soil electrical conductivity was reduced by 39.16% relative to untreated soil, and wheat plant height, chlorophyll content, fresh/dry weights, and nitrogen uptake were significantly enhanced, confirming the effectiveness of structural design strategies for developing agricultural water-retaining agents [84].

The combination strategy integrates multiple methods to achieve synergistic enhancement. For instance, the combination of hydrophobic encapsulation and solvent optimization, such as the PDMS/glycerol system, can achieve a weight loss of less than 2% over a wide temperature range of −20 °C to 80 °C [85]. Additionally, the combination of ionic incorporation and nanofillers simultaneously enhances conductivity and antifreeze properties [86]. This strategy maximizes water retention efficiency by coupling multiple mechanisms, including hydrogen bonding, ionic hydration, and steric hindrance. However, it requires optimizing component ratios to avoid interface incompatibility or functional conflicts [86]. The combinatorial strategy provides agricultural water-retaining agents not merely with the performance labels of “broad temperature tolerance and high water retention,” but also with a modularly assemblable hydrogel design framework. Depending on target agricultural scenarios—such as seedling protection in cold regions, saline–alkali soil remediation, dryland seeding, and synergistic water–fertilizer controlled release—researchers can flexibly select and combine strategies, including hydrophobic encapsulation, solvent optimization, ion incorporation, and nanoreinforcement, to construct customized hydrogel carriers. The true agricultural value of this strategy lies not in simply replicating materials-science systems such as PDMS/glycerol or MXene, but in applying its “multi-mechanism coupling” design philosophy to achieve enhanced water retention while ensuring the hydrogel’s ecological safety.

**Table 1:** Summary of representative material systems and quantitative environmental tolerance performance indicators in different modification strategies of hydrogels.

Modification Strategy	Typical Material System	Functional Mechanism	Quantitative Performance
Encapsulation Strategy	PDMS (polydimethylsiloxane) hydrophobic coating	Constructs a physical/chemical barrier on the hydrogel surface to block vapor permeation directly	Retains 80.45% of initial weight within 72 h; non-encapsulated counterpart retains only 8.94% [72,73]
Solvent Optimization	Glycerol/aqueous system	Polyol molecular clusters trap free water and depress vapor pressure	Endows hydrogel with antifreeze ability and enhanced self-healing performance
Solvent Optimization	Ethylene glycol (EG)/water system	Alters the hydrogen-bond network to inhibit ice nucleation	Maintains excellent tensile strength over −60 °C to 60 °C [75]
Solvent Optimization	PAA-CNF-HILs ionic gel	Hydrated ionic liquids with ultra-low volatility	Broadened working window of −45 °C to 75 °C [76]
Ionic Incorporation	CaCl_2_-incorporated hydrogel	Primary/secondary hydration shells disrupt the water H-bond network	Withstands temperatures down to −58 °C [78]
Ionic Incorporation	LiCl-incorporated hydrogel	Hygroscopic ionic hydration converts free water into bound water	Operates down to −80 °C with self-regeneration from ambient humidity [80]
Structural Design	CPAUH multifunctional hydrogel (CNF + p(AA-co-KAA))	CNF represents a rigid network, while p(AA-co-KAA) represents a flexible network	Mechanical strength: 0.169 MPa. Absorption capacity: 121.65 g/g. After three cycles, it still reached 118 g/g. After loading with urea and humic acid, the cumulative leaching rates within 15 days were only 66.91% and 92.45%, respectively. When applied to saline–alkali soil, the soil conductivity decreased by 39.16% [84].
Combination Strategy	PDMS/glycerol hybrid system	Interfacial blocking + thermodynamic water-state locking	Weight loss < 2% across a wide range of −20 °C to 80 °C [85]
Combination Strategy	Ionic doping + nanofiller composites	Coupled ionic hydration and steric confinement	Simultaneously boosts conductivity stability and freezing tolerance [86]

### 3.4. Application of Hydrogels in Controlled-Release and Sustained-Release Fertilizers

Hydrogels can also be used in controlled-release fertilizers to achieve water–fertilizer synergy and promote the integration of water and nutrient management. This approach not only significantly improves fertilizer utilization and reduces the environmental impact of nutrient loss but also enhances soil moisture conditions, improves crop drought resistance, and effectively reduces fertilization frequency and labor costs [87]. In current agricultural applications, hydrogels are increasingly used as carriers for fertilizers and nutrients. By encapsulating nutrients within its polymer network [88], intelligent nutrient-controlled-release hydrogels can be constructed to respond to actual soil moisture levels, enabling on-demand nutrient release. Compared with traditional fertilization methods, this system not only significantly improves fertilizer utilization but also effectively reduces nutrient loss, thereby alleviating the common environmental burdens associated with conventional practices [89]. Furthermore, nutrient-loaded hydrogels can control the release rate of specific nutrients according to the nutritional demands of crops at different growth stages, ensuring that plants receive an appropriate nutrient supply during critical growth periods [67,90].

Hydrogels, leveraging their three-dimensional polymer networks and tunable pore structures, can precisely regulate the release rate of encapsulated active molecules via mechanisms such as Fickian diffusion, thereby avoiding the burst-release effect and serving as ideal carriers for the sustained and controlled release of substances. In the field of agri-food active packaging, this capability is particularly important. For example, although plant essential oils possess excellent antibacterial and antioxidant properties, their high volatility and strong odor mean that direct application results in rapid loss of active ingredients and uncontrolled release. By encapsulating them in a hydrogel matrix, the escape of volatiles can be delayed through multiple mechanisms, including hydrogen bonding, hydrophobic interactions, and physical network entrapment, thereby keeping the effective concentration in the packaging headspace consistently above the minimum inhibitory dose. Researchers used cellulose extracted from grape-stalk waste as the base material and Salep as a copolymer to fabricate a hydrogel, which was then freeze-dried into a dense bioaerogel that served as a controlled-release carrier for Zataria multiflora essential oil (ZMEO) in the modified-atmosphere packaging of ground beef. The ZMEO active components released into the headspace of the package eliminated microorganisms by destroying bacterial cell membranes; meanwhile, the phenolic compounds in ZMEO delayed the oxidation of lipid-rich foods by scavenging free radicals, reducing Fe^2+^/Cu^2+^-catalyzed Fenton reactions, and lowering malondialdehyde (MDA) production. GC-MS analysis indicated that carvacrol and thymol accounted for 55.29% and 19.63% of ZMEO, respectively; headspace antimicrobial tests determined its minimum inhibitory dose against native meat microbiota and Pseudomonas aeruginosa to be 256 μL/L headspace. In storage experiments, after 9 days at 7 °C, the cellulose aerogel loaded with 10×MID ZMEO reduced total mesophilic bacteria in ground beef by 1.8 log_10_ CFU/g, total psychrotrophic bacteria by 2 log_10_ CFU/g, and total coliforms by 1.4 log_10_ CFU/g (the control group exceeded the TVN limit on day 6, whereas the treated group only reached 18.9 mg/100 g TVN by day 9) [91]. This work not only confirmed the effectiveness of the hydrogel carrier in the sustained release of volatile antibacterial agents but also demonstrated the significant value of using agricultural waste to develop sustainable controlled-release hydrogel packaging materials.

A critical limitation of existing studies is their confinement to idealized laboratory conditions, which overlooks the complex interferences present in field environments, such as fluctuations in temperature, pH, ionic strength, microbial activity, and root exudates. Most studies merely measure cumulative release rates without simultaneously monitoring the actual nutrient uptake dynamics of crops. In the context of food packaging, the storage stability of essential-oil active components and the safety of their degradation products, as well as the migration compliance of hydrogels used as food-contact materials, are frequently neglected. Furthermore, the economic feasibility of expensive processing techniques such as freeze-drying and the use of rare raw materials like Salep remains unexplored. Considerable heterogeneity exists across studies in release media, temperature, and sampling frequency, and the absence of standardized evaluation criteria precludes meaningful cross-study comparisons. Future research should systematically assess hydrogel performance under realistic conditions and establish widely recognized performance standards.

### 3.5. Three Forms of Hydrogels for Achieving Controlled-Release Fertilizers

Membrane Material Hydrogel

The coating material hydrogel achieves this by enclosing the fertilizer particles with a protective layer of thermosensitive or smart-responsive hydrogel. The hydrogel’s three-dimensional network structure acts as a physical barrier against water penetration and nutrient diffusion, thereby enabling controlled release. The coating layer of this type of hydrogel can undergo reversible swelling or contraction in response to external stimuli such as temperature and pH, thus regulating the rate of nutrient release. For example, the functional dual-layer coated fertilizer DCRF developed by Shandong Agricultural University (Jinan, China) has an outer layer formed by cross-linking sodium alginate with copper ions to create a hydrogel membrane, and an inner layer made of biobased polyurethane. The dual-layer structure works together to delay the release of nitrogen nutrients, and at the same time, the copper ions in the outer layer can be slowly released to inhibit soil-borne diseases [92,93]. In the early stage, due to the presence of unbound copper ions in the hydrogel network, the release rate of copper ions is relatively fast. In the later stage, the release rate of copper ions is slower because of the binding of copper ions with sodium alginate. The release of copper ions is primarily driven by the decomposition of the hydrogel. The slow release of copper ions provides sustained antifungal ability for the fertilizer, effectively preventing fungal diseases from infecting the soil throughout the entire growth period, thereby allowing plants to grow healthily [93].

Within the domain of food active packaging, alginate-based coating hydrogels have been widely adopted. For instance, researchers developed edible films using sodium alginate (SA) and carboxymethyl cellulose (CMC) as matrix materials, incorporating 0.1%, 0.2%, and 0.3% cinnamaldehyde (CN) as a controlled-release carrier to preserve marzipan. Material characterization revealed that with increasing CN concentration, film opacity increased significantly from 1.64 to 29.58 A/mm, and the water vapor permeability rose from 0.18 to 0.44 g·mm/(m^2^·h·kPa); concurrently, the tensile strength of the 0.3% CN film decreased markedly while the elongation at break increased to 13.50%. Fourier-transform infrared (FT-IR) spectroscopy exhibited the characteristic C=O stretching vibration of CN at 1735 cm^−1^, confirming intermolecular interactions between CN and the SA–CMC matrix, which provided the requisite structural basis for CN sustained release. Antimicrobial assays demonstrated a positive concentration-dependent response, with the 0.3% CN film producing an inhibition zone against Escherichia coli reaching 17.41 mm. Following a 50-participant sensory evaluation, the 0.2% CN-coated group achieved the highest scores for both “willingness to eat” and “willingness to purchase”; accordingly, this concentration was selected for the formal storage trial. Results from storage at 25 °C for 15 days indicated that the 0.2% CN coating reduced the total aerobic mesophilic count in marzipan from 3.39 to 2.87 log CFU/g and decreased the peroxide value from 6.46 to 5.76 meq O_2_/kg, whereas no statistically significant improvement was observed in free fatty acid levels [94]. This work substantiates the efficacy of SA–CMC coating matrices in retarding the release of the volatile antimicrobial cinnamaldehyde and highlights the utility of polysaccharide-based hydrogels in advancing sustainable controlled-release packaging paradigms.

Matrix-type hydrogels

**Figure 3:**
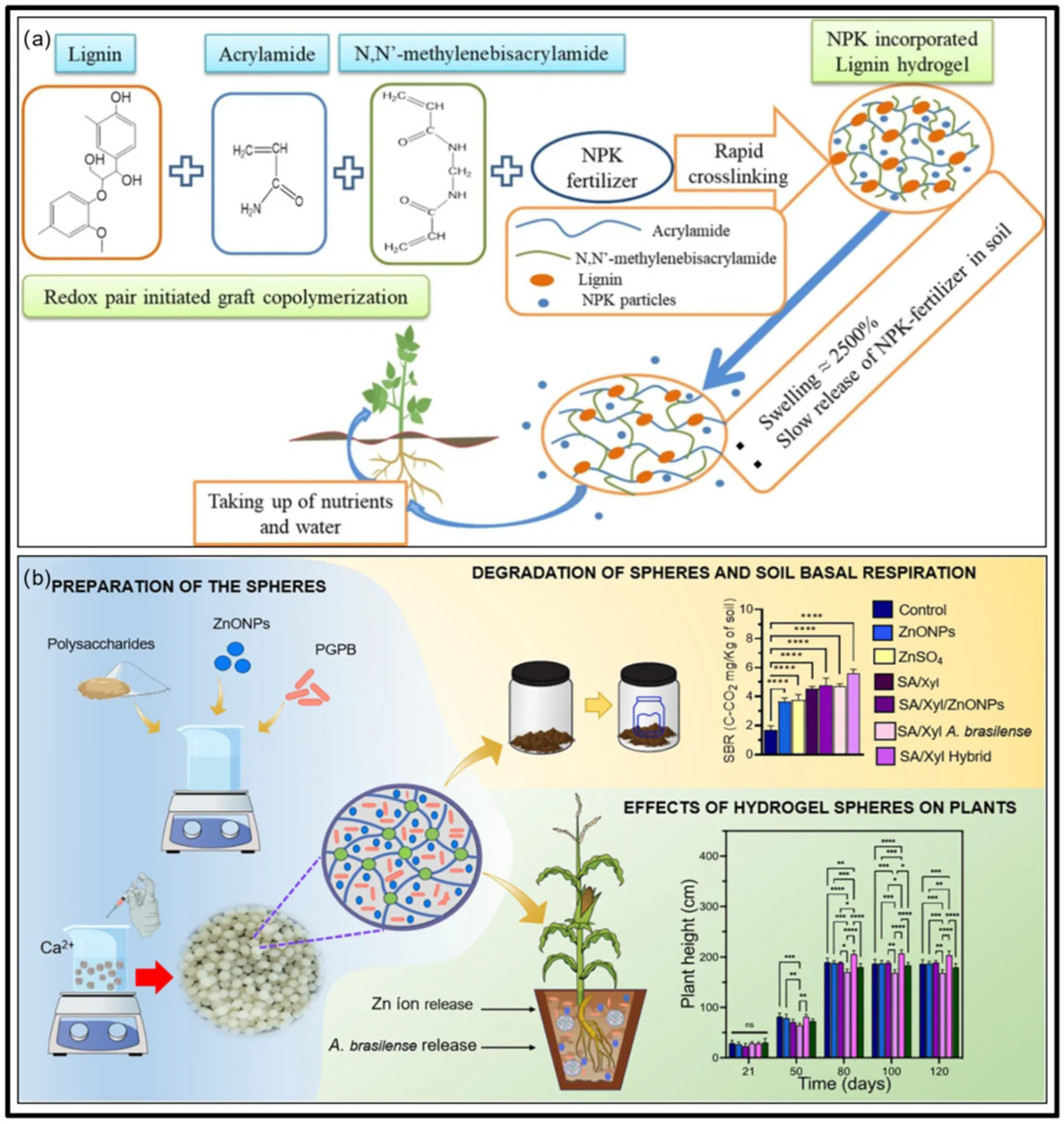
(**a**) Construction of redox-driven lignin-based hydrogels and their NPK fertilizer slow-release. Reproduced with permission from reference [97]. (**b**) Development of mixed hydrogel spheres based on sodium alginate (SA) and xylan (Xyl), for simultaneous delivery of ZnO nanoparticles and promotion of plant growth by the dual controlled release of bacteria from Brazilian algae. *, **, ***, and **** indicate statistical significance at *p* < 0.05, *p* < 0.01, *p* < 0.001, and *p* < 0.0001, respectively; ns denotes not significant (*p* ≥ 0.05). Reproduced with permission from reference [98].

Matrix-type hydrogels uniformly disperse or adsorb fertilizer molecules within their three-dimensional network, and nutrients are released slowly through the swelling-diffusion process of the hydrogel [95]. The release kinetics of these hydrogels are regulated by the cross-linking density, pore structure, and environmental osmotic pressure. These hydrogels typically use natural or synthetic polymers as the framework, with the fertilizer serving as the filler phase within the network [96]. For example, in Figure 3a, the researchers synthesized base lignin (AL) by using acrylamide (AM) as the monomer, N,N′-methyl bisacrylamide (MBA) as the crosslinking agent, and a new oxidative-reductive initiation system consisting of ammonium persulfate (APS) and ferrous sulfate (FS). They also prepared AM-g-AL-MBA hydrogels. These hydrogels achieved a porosity of 72% and released 43% of the fertilizer within 14 days. They also demonstrated significant advantages in maintaining moisture and efficient nutrient delivery under non-Fickian diffusion mechanisms [97]. The hydrogels can not only carry traditional fertilizers but also microbial agents such as bacteria. For example, in Figure 3b, the researchers used natural polysaccharides such as sodium alginate and xylan as the matrix and formed biodegradable hydrogel microspheres by crosslinking with Ca^2+^ ions, which encapsulate ZnO nanoparticles and beneficial bacteria. This can simultaneously achieve the slow release of trace nutrients and the high survival delivery of beneficial bacteria. The encapsulation significantly prolonged the bacterial survival time, the viable cell count reached 7.17 × 10^7^ CFU/mL (SA/Xyl) after 1400 h, while the free bacteria were only 2.9 × 10^4^ CFU/mL. They have good application prospects in reducing fertilizer usage and improving soil microecology. The SA/Xyl hybrid microsphere treatment group promoted the development of corn root systems and increased the plant’s absorption of zinc (Zn uptake), demonstrating the synergistic potential of micro-nutrient supply and biological stimulation [98]. Cellulose derivative hydrogels, such as hydroxypropyl methylcellulose, can affect nutrient diffusion pathways through the movement and phase behavior changes in their chain segments. Their sustained-release effect is closely related to the glass transition temperature of the polymer chain [99]. Agarose-based composite hydrogels, used as the matrix, load seleniumated carrageenan or sodium selenite for selenium-enriched cultivation of vegetable seedlings. They maintain the stability of the gel structure while achieving a continuous supply of selenium and retain selenium enrichment ability even after four cycles of use [100,101].

Composite hydrogels

Composite hydrogels combine hydrogels with other functional materials to construct multilevel structures or synergistic systems [102]. While enhancing mechanical properties, environmental stability, and intelligent responsiveness [103], these composite components introduce multiple controlled-release mechanisms such as adsorption–desorption, ion exchange, or photocatalytic degradation, thereby allowing the release rate and duration of the substances to be extended and controlled as needed [104,105]. For example, sodium alginate hydrogels doped with natural soil colloids or nanoscale TiO_2_ not only increase the hydrogel’s thermal decomposition temperature by approximately 20 °C but also extend nutrient diffusion time by filling the network pores with nanoparticles [106]. Combining hydrogels with biochar utilizes biochar to adsorb fertilizers, followed by regulation of release through the swelling response of the hydrogel, achieving a dual “adsorption–sustained release” control mechanism [107,108]. Additionally, combining metal–organic frameworks, such as MIL-100(Fe), with cellulose/sodium alginate hydrogels allows the high specific surface area and tunable pore structure of the framework to further finely control the release rate of nutrients [109].

Significant shortcomings persist in the aforementioned studies on the three forms of hydrogels. First, membrane-type hydrogels enable stimulus-responsive release, but the long-term integrity of their coatings under repeated wetting–drying cycles and mechanical abrasion in field conditions has not been validated. Moreover, the antibacterial efficacy of sustained copper ion release has only been demonstrated under laboratory conditions, without accounting for the immobilization or antagonistic effects of soil microorganisms on copper ions. Second, the release kinetics of matrix-type hydrogels are predominantly fitted using pure water systems, neglecting the interference of competitive binding among multiple ions in soil solutions on diffusion behavior. Meanwhile, the live-cell delivery efficiency of microbial carrier hydrogels may be substantially reduced in real soil due to competition and predation by indigenous microbial communities, and existing survival data cannot adequately represent field performance. Third, composite hydrogels incorporating nanofillers or metal–organic frameworks (MOFs) prolong release duration, yet the potential ecotoxicity, degradation product fate, and large-scale production costs of these materials remain largely undiscussed. Furthermore, there is a lack of horizontal comparison among the three forms, making it impossible to guide users in selecting the optimal formulation based on soil type, crop requirements, and cost budget. Future research should establish a cross-form unified evaluation framework and validate the practical efficacy and sustainability of these hydrogels under field conditions.

### 3.6. Fertilizer Diffusion in Hydrogels: Three Mechanisms

Fick diffusion is the fundamental mass transfer mechanism for nutrient release in hydrogels, and the release process is primarily governed by physical diffusion [110]. The kinetics of nutrient release in most hydrogel systems conform to the Fick diffusion model. When the hydrogel absorbs water and swells, numerous water-filled micropores form within its three-dimensional polymer network, providing channels for nutrient migration [35,111]. The dissolved fertilizer ions or small molecules, driven by concentration gradients, spontaneously diffuse from the interior of the hydrogel to the surrounding soil solution. This process is similar to the diffusion behavior of molecules in a homogeneous medium, and its rate is mainly influenced by the pore size of the hydrogel network, the tortuosity of the diffusion path, and the concentration gradient of the nutrient [87]. For example, in biochar-modified hydrogels, the binding of nutrients to the hydrogel is enhanced through hetero-interface electron transfer; however, nutrient release is still primarily driven by the concentration gradient-based mechanism [111].

The swelling-controlled diffusion mechanism endows hydrogels with intelligent, environment-responsive release characteristics. The swelling behavior of such stimulus-responsive hydrogels can be regulated by external environmental conditions such as temperature, pH, and ionic strength [112,113]. For instance, the typical thermosensitive material poly(N-isopropylacrylamide) (PNIPAM) exhibits a lower critical solution temperature (LCST). When the environmental temperature is below the LCST, the hydrogel network becomes hydrophilic and swells, opening the channels and allowing nutrient diffusion. Conversely, when the temperature rises above the LCST, the network undergoes a hydrophilic-hydrophobic transition, rapidly contracting and expelling water along with the loaded nutrients, thereby significantly increasing the release rate [114]. This property enables a positive correlation between nutrient release and crop growth temperature, thereby enhancing the temporal and spatial matching of nutrient utilization.

The chemical bonding-controlled diffusion mechanism achieves long-term, slow nutrient release through chemical interactions [115]. In this design, fertilizer molecules are not physically embedded but are covalently grafted onto the polymer backbone of the hydrogel via breakable chemical bonds, such as ionic bonds, ester bonds, and amide bonds. The release of nutrients depends on the gradual degradation of these chemical bonds through hydrolysis reactions or microbial enzyme catalysis within the soil environment [116]. Because the rate of chemical bond breakage is relatively constant and slow, this mechanism can achieve near-zero-order release kinetics. It avoids the phenomenon of excessive nutrient release during the initial stage and provides a stable, continuous nutrient supply, making it suitable for meeting the nutrient demands of crops throughout their entire growth period [117].

This article enumerates three mechanisms of fertilizer diffusion in hydrogels, along with examples of their applications, but it lacks a critical examination of their inherent limitations and the conditions under which they are applicable. For instance, the Fickian diffusion model assumes a homogeneous medium yet fails to account for deviations arising from the heterogeneity of the hydrogel network. The near-zero-order kinetics claimed for chemically bonded controlled release are susceptible to disruption by fluctuations in soil enzyme activity and pH. Moreover, the synergistic or competitive effects among different mechanisms have not been explored. A more systematic evaluation of the release kinetics of hydrogels under multi-factor coupling in actual soil environments is still needed.

### 3.7. Application of Hydrogels in Heavy Metal Adsorption

**Figure 4:**
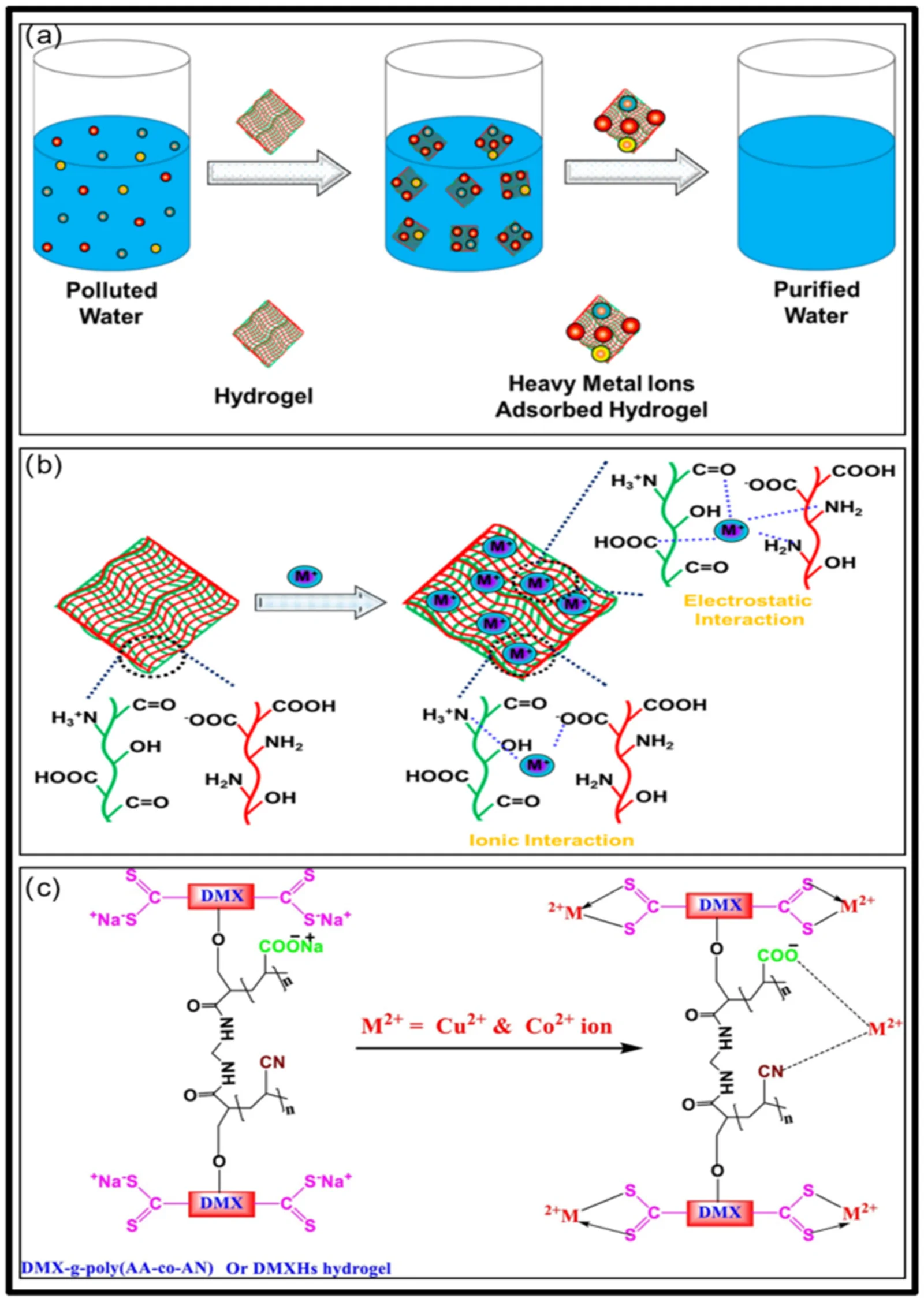
(**a**) Schematic diagram of the hydrogel adsorbent removing heavy metal ions from contaminated water. (**b**) Schematic diagram of the mechanisms involved in the removal process of heavy metal ions. Reproduced with permission from reference [122]. (**c**) Possible mechanisms for the adsorption of Cu^2+^ and Co^2+^. Reproduced with permission from reference [121].

Hydrogels have unique application potential for adsorbing heavy metals in agricultural environments and ensuring global food security. In practical applications, hydrogels can be directly applied to contaminated soil, where they adsorb and fix the heavy metals and are subsequently collected and treated [118]. Some studies have also used hydrogels to construct permeable reactive barriers or to treat agricultural irrigation water [119]. The functional groups contained in hydrogels, such as carboxyl (-COOH), amino (-NH_2_), and sulfonic acid (-SO_3_H), enable efficient and selective capture of specific heavy metal ions through various chemical adsorption mechanisms, including chelation, coordination bonding, and ion exchange. These mechanisms work in synergy with processes such as electrostatic attraction and physical adsorption [120,121]. Figure 4 illustrates the key mechanisms and performance of hydrogels in heavy metal adsorption applications, including the adsorption process, synthesis scheme, and specific removal mechanisms [122].

Figure 4c illustrates the proposed mechanism for the adsorption of Cu^2+^ and Co^2+^ ions onto the DMXHGs-3 hydrogel surface. It is clearly mentioned that the cross-linked network of DMXHGs-3 hydrogel contains numerous functional groups such as -COO^−^, -C(=S)S^−^, -CN, and -OH, which act as better ligands and may also capture Cu^2+^ and Co^2+^ ions through physical and chemical interactions, as clearly mentioned in Figure 4c [121]. The adsorption capacity of hydrogels for heavy metal ions is also influenced by the environmental acidity and alkalinity, as well as the self-swelling property of the hydrogels. For instance, the polyvinyl alcohol/xanthan gum (PVA/XG3) hydrogel can adsorb methylene blue (MB) and heavy metal ions such as Pb^2+^ in wastewater. However, the swelling degree of the PV/AXG3 hydrogel decreases under acidic conditions. Compared with alkaline conditions, the adsorption performance of the PVA/XG3 hydrogel for MB and Pb^2+^ also decreases under acidic conditions [123]. Additionally, the adsorption of Cr(VI) and Hg(II) by the composite hydrogel for capture is affected by pH: studies have shown that under pH ≥ 2 conditions, the Cr(VI) anion usually exists in the form of HCrO_4_^−^. The negatively charged HCrO_4_^−^ ions are easily adsorbed onto the protonated hydrogel matrix through electrostatic interactions, indicating that the composite hydrogel can provide a basis for the high-sensitivity detection of heavy metal ions [121]. The heavy metal adsorption behavior of hydrogels is governed by their functional group composition, leading to substantial variations in selective adsorption efficiency among different materials. In soil environments, coexisting ions such as Ca^2+^ and Mg^2+^ may compete with target heavy metals for adsorption sites, potentially leading to suboptimal hydrogel performance in field applications. Furthermore, the regeneration and recyclability of hydrogels have rarely been discussed, and the high cost of synthesis and recovery poses significant challenges to their large-scale deployment.

### 3.8. Application of Hydrogel in Pesticide Residues

In modern agriculture, pesticides are crucial for ensuring food security, increasing yield, and maintaining the quality and efficiency of agricultural products. Pesticides not only prevent pests and diseases and help recover yield losses but also ensure the appearance and intrinsic quality of agricultural products. However, when pesticides are used improperly, they can lead to excessive residues, causing a series of problems [124,125]. Excessive pesticide residues pollute the environment, contaminate soil and water bodies, and secondarily contaminate crops through these media, thereby affecting the sustainable development of agriculture. Furthermore, long-term intake of trace pesticide residues may damage the liver, kidneys, and nervous system, increase the risk of chronic diseases, and pose even greater threats to vulnerable populations such as children and pregnant women [126,127]. Thus, hydrogel is a possible solution to cope with pesticide residues from contaminated water and soil, minimize secondary pollution, and reduce environmental drought persistence, thereby increasing crop growth and development.

**Figure 5:**
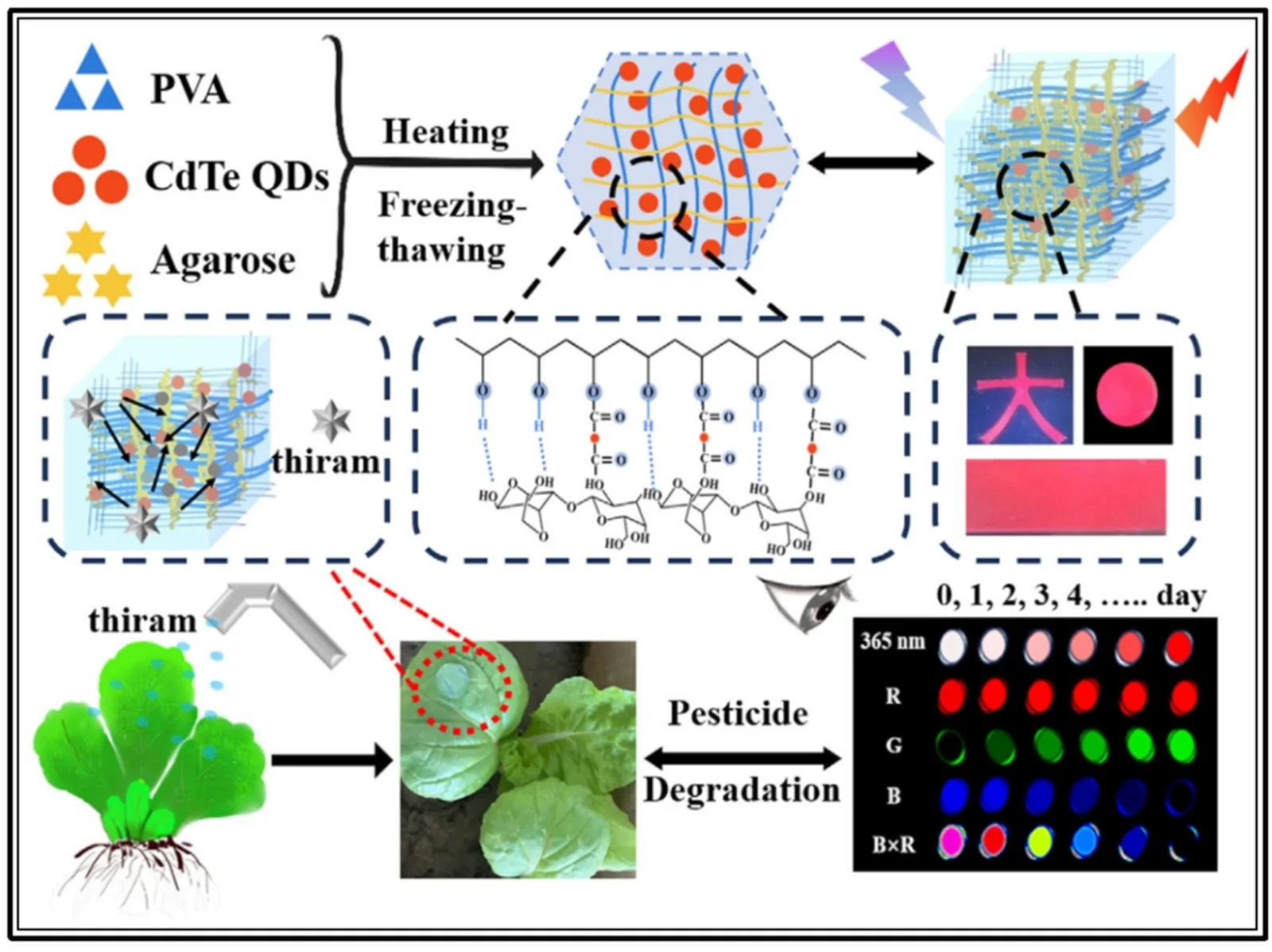
Schematic diagram of the preparation of CdTe CDs@PVA@AG for monitoring the dynamic degradation of pesticides in an undamaged manner. Reproduced with permission from reference [132]. R, G, B, and B × R indicate red, green, brown, and brown × red. Copyright 2025 Wiley-VCH GmbH.

The numerous functional groups present in the hydrogel network can bind to pesticide molecules through hydrogen bonds, electrostatic interactions, and hydrophobic forces, thereby immobilizing them within the network structure. This reduces the amount of pesticides required and lowers pesticide residues in the environment [128,129]. For instance, researchers have developed a cross-linked hydrogel (CLPHMAs) based on polyvinyl alcohol (PVA) as the backbone and using lactic acid as an environmentally friendly crosslinking agent. This cross-linked hydrogel can effectively adsorb pesticides such as carbofuran and methomyl in water. Under optimal conditions, the capture amounts of both pesticides reached approximately 0.064 mg/mg, demonstrating a significant removal effect [130]. By combining the hydrogel with photocatalytic materials, pesticides and agricultural pollutants can be adsorbed and then decomposed into harmless substances using light energy. For example, integrating the photocatalyst CuS@MIL-100 into a cellulose hydrogel achieved a degradation efficiency of 93.3% for the typical organic pollutant tetracycline (an antibiotic) [131]. Furthermore, hydrogels can be fabricated into sensors for detecting pesticide residues. For instance, embedding CdTe quantum dots (QDs) into a dual-network hydrogel assembled from polyvinyl alcohol (PVA) and agarose (AG) forms a wearable fluorescent sensor. This sensor utilizes the red fluorescence and stimulus-responsive properties of CdTe QDs, combined with the flexibility, high adhesion, and porous structure of the hydrogel, to enable on-site rapid detection of pesticide residues [132]. As illustrated in Figure 5, the flexible and portable fluorescence hydrogel detector (CdTe QDs@PVA@AG) exhibits bright red emission and a selective response to thiram. The fluorescence hydrogel was composed of thiram-responsive CdTe QDs, PVA, and AG, and cross-linked via a freeze–thaw process. Harnessing the red emission of CdTe QDs, the abundant porosity, as well as the high transparency of the AG–PVA hydrogel, CdTe QDs@PVA@AG was cast and solidified in a mold as a fluorescence hydrogel detector for thiram, which enhanced the operability, stability, and antifouling properties. The CdTe QDs@PVA@AG hydrogel exhibits excellent flexibility and adheres firmly to the surfaces of crops and fruits. Combined with light excitation and smartphone-assisted imaging and data-processing capabilities, CdTe QDs@PVA@AG enables in situ and on-site analysis of thiram residues. Interestingly, thiram molecules freely diffuse through the hydrogel’s 3D polymer network due to its intrinsic permeability, thereby allowing more target molecules to interact with CdTe QDs and modulate the output signal, significantly improving detection sensitivity.

Hydrogels have been demonstrated to be effective for pesticide residue management. That said, their long-term stability and reusability may be affected in practical applications by complex field factors—such as competing organic matter, fluctuating pH levels, and the microbial degradation of the hydrogel itself. Moreover, whether degradation byproducts, particularly those derived from photocatalytic composites such as CuS@MIL-100, are toxic remains unknown.

### 3.9. Application of Hydrogels in Precision Agriculture

Hydrogels, with their unique three-dimensional network structure and intelligent response characteristics, play an important role in precision agriculture [6]. In the areas of intelligent sensing and plant monitoring, hydrogels can be used to monitor the health status of plants in real time. For example, self-powered hydrogel sensors can be directly attached to plant leaves. By monitoring changes in the relative water content of the leaves, these sensors enable non-invasive determination of plant water stress conditions. When plants are under drought stress, the output voltage of the sensor will decrease, while in waterlogging conditions, the output voltage increases abnormally. These data are uploaded to the cloud via the Internet of Things system, providing a direct basis for precise irrigation [133]. Additionally, a research team at Jilin University developed a hydrogel-based fluorescence sensor for on-site rapid detection of chlorpyrifos pesticide residues on Chinese cabbage and for monitoring its degradation process, achieving a detection limit as low as 0.2 ng/mL. This provides an effective tool for the precise and safe application of pesticides [134].

In water and fertilizer precision management, hydrogels can serve as effective carriers. Some intelligent gels can precisely regulate the release of water and fertilizer in response to environmental changes and crop needs, enabling climate-smart slow-release and on-demand supply [5,135]. For example, agricultural waste straw was converted into a pH-responsive intelligent hydrogel fertilizer. This hydrogel can intelligently regulate nutrient release according to the soil pH, achieving a cumulative release rate of less than 75% within 28 days. This not only improves nutrient utilization efficiency but also realizes the resource utilization of agricultural waste [136].

To translate the multiple demonstrated application cases of hydrogels in precision agriculture into practical scenarios, numerous challenges must be addressed. For instance, the durability and signal stability of self-powered sensors in complex field environments have yet to be validated; fluorescent sensors exhibit low detection limits, but their anti-interference capability and long-term storage performance remain undiscussed. Furthermore, issues such as whether the release behavior of pH-responsive hydrogel fertilizers is consistent across different soil types and whether the production and usage costs of hydrogels are controllable remain prevalent.

## 4. The Effects of Hydrogels on Plant Growth

### 4.1. Effects of Hydrogels on Soil Structure Improvement and Plant Root Systems

When hydrogels are mixed with the soil, the active groups on their molecular chains, such as carboxyl and hydroxyl groups, form a network through the adhesive effect. This network connects dispersed soil particles, forming aggregates and thereby optimizing the soil’s physical structure. Some specially designed hydrogels can also enhance the mechanical stability of the soil [137]. For example, a bio-inspired hydrogel developed by Beijing University of Technology can increase the compressive strength of the stabilized soil to 1–2.9 MPa, effectively preventing soil erosion and sedimentation [137]. After hydrogels are added to the soil, the proportion of water-stable aggregates larger than 0.25 mm can increase by 30%. This aggregate structure effectively increases soil porosity, enhances air permeability, and facilitates root expansion [138,139].

The significant promotion of plant root development is one of the core functions of hydrogels on plant root and shoot growth, yield-related traits, water uptake via membrane stability, nutrient uptake and signaling, as well as beneficial root–microbe interactions [140]. The adsorption and slow-release mechanism of hydrogels applies to both water and nutrients, effectively regulating the microenvironment in the root zone [141]. Due to their strong water absorption and water retention capacity, hydrogels can absorb hundreds to thousands of times their weight in water during rainfall or irrigation and store it. They then release the water slowly as the soil dries, maintaining a relatively stable moist environment while improving soil aeration and structure, thereby creating more suitable conditions for root and shoot growth [142]. Hydrogels can also fix substances such as urea and microbial fertilizer within their three-dimensional network and release them slowly. Compared with traditional fertilization methods, in which approximately 70% of the nitrogen fertilizer is lost to volatilization, the use of hydrogels significantly reduces nutrient loss and improves fertilizer utilization. This continuous, stable supply of water and nutrients promotes the development of more extensive root systems [143]. Under stressful conditions such as drought or heavy metal pollution, hydrogels help plants retain water and enhance stress resistance through special mechanisms [144,145]. For example, in chromium-contaminated soil, chitosan–Bacillus subtilis composite hydrogel microspheres can help lettuce reduce chromium absorption by adsorbing and inactivating chromium ions and by activating the plant’s antioxidant enzyme system [146].

### 4.2. Effects of Hydrogels on Improving Plants’ Drought Resistance and Salt–Alkali Tolerance

#### 4.2.1. Enhancing Plants’ Drought Resistance

Hydrogels can quickly absorb and retain hundreds to thousands of times their weight in water, releasing it slowly during soil drying, thereby prolonging the effective water supply time for plants [147]. The water retention capacity of hydrogels ensures that plants can obtain water even when external water sources are scarce, thus improving water-use efficiency. For example, researchers developed an environmentally friendly hydrogel based on carboxymethyl cellulose (CMC) and systematically evaluated its promoting effect on wheat seed germination and seedling growth under drought stress. Under severe drought conditions with a field moisture content of 40%, treatment with 2% hydrogel significantly increased the germination rate of wheat seeds from approximately 45% in the control group to over 70%, representing an increase of more than 25% [148]. In sub-Saharan regions, drought is a major challenge. In a study conducted in Kenya, researchers applied super-absorbent hydrogels to corn fields to assess their impact on water retention and crop yield. The results show that the soil moisture level in the treated fields was 25% higher than that in the untreated fields, and the corn yield in the dry season increased by 20%, highlighting the potential of hydrogels in drought-prone regions [149].

Hydrogels can markedly improve plant drought resilience through a multilevel cascade mechanism bridging material design, rhizospheric soil modulation, and plant physiological adaptation. For instance, the biochar-composite hydrogel S-B-AA effectively enhances the drought adaptability of tobacco. At the material level, the incorporation of biochar enriches the spatial pore-network architecture of the hydrogel matrix and introduces abundant hydrophilic active sites—such as –OH and –COOH groups—thereby elevating its equilibrium swelling degree by 44.7% relative to the single-network hydrogel S-AA. This enhanced water-retention capacity translates into a significant 17.1% increase in the maximum soil water content compared with the control, thereby establishing a functional “micro-reservoir” in the root zone. At the plant physiological level, S-B-AA treatment significantly increases total leaf water content, relative water content (RWC), and the free water proportion, while concurrently reducing bound water content and water saturation deficit (WSD). Improved hydration status allows stomata to remain open under drought stress, thereby elevating net photosynthetic rate, stomatal conductance, and transpiration rate, and enhancing PSII photochemical parameters (Fv and actual quantum yield, QY), ultimately facilitating aboveground biomass accumulation. Meanwhile, sustained moisture buffering effectively suppresses drought-induced overproduction of superoxide anions (O^2−^) and hydrogen peroxide (H_2_O_2_), thereby reducing malondialdehyde (MDA) accumulation by 28.4%. Correspondingly, the activities of major antioxidant enzymes (SOD, POD, and CAT) and endogenous H_2_S signaling molecules are synchronously downregulated, indicating that plants maintain plasma membrane integrity without mobilizing exhaustive oxidative stress defenses. From the perspective of hormonal regulation, S-B-AA decreases foliar abscisic acid (ABA) and gibberellin (GA_3_) levels by 81.3% and to below the limit of detection, respectively, mitigating the hyperactivation of drought-triggered hormonal signals. Transcriptomic profiling further corroborates these findings: compared with the control, S-B-AA upregulated 2826 and downregulated 5225 differentially expressed genes (DEGs). GO and KEGG enrichment analyses revealed that these DEGs are predominantly concentrated in the MAPK signaling pathway, plant hormone signal transduction, and photosynthesis-related pathways. Notably, genes encoding chloroplast thylakoid membrane components, photosystem I/II core subunits, and light-harvesting antenna proteins exhibited pronounced transcriptional remodeling. In summary, the S-B-AA hydrogel orchestrates a systems-level enhancement of tobacco drought tolerance—spanning from phenotypic performance to transcriptomic reprogramming—through the synergistic integration of root-zone soil moisture amelioration, photosynthetic and osmotic optimization, ROS storm mitigation, and recalibration of hormonal and MAPK signaling networks [150].

#### 4.2.2. Enhancing Plant Salt–Alkali Tolerance

Hydrogels reduce the effectiveness of salt ions (such as chloride ions) in soil through physical adsorption, improve water retention regulation, and directly or indirectly promote crop growth [151]. For instance, using synthetic biochar combined with PEtOx-HEMA-CS composite hydrogels (poly(2-ethyl-2-oxazoline)/poly(2-hydroxyethyl methacrylate)/chitosan as a soil amendment significantly improved the physical and chemical properties of saline–alkali soil. This treatment not only reduced soil salinity and the percentage of exchangeable sodium but also enhanced water retention capacity, organic matter, and cation exchange capacity. The relative increase rates of nitrogen, phosphorus, and potassium contents in the root of the carrot are 36.36%, 70%, and 72%, respectively (*p* < 0.05). Additionally, the relative growth rate of carrot yield increased by 49.63% [152]. Acrylic acid (AA) and acrylamide (AM) were added to ultrapure water, and then XLG was added and stirred for dispersion. After that, N,N′-methylene bis(acrylamide) (MBA) and ammonium persulfate (APS) were added and heated to initiate the formation of P(AA-co-AM)/XLG hydrogel. To deal with the problem of cadmium (Cd) contamination in saline–alkali soil, the P(AA-co-AM)/XLG hydrogel was dried and then added to the Cd-contaminated saline–alkali soil, and Suaeda salsa was planted on the surface. Conversely, the impact of the P(AA-co-AM)/XLG hydrogel on Suaeda salsa was further investigated, as well as the influence of both the physicochemical properties and microbial communities of the saline–alkali soil. The P(AA-co-AM)/XLG composite hydrogels prepared by free radical polymerization using AA and AM as monomers and Laponite XLG as a nanofiller could not only adsorb and fix heavy metal Cd in the soil but also synergistically remediate the combined pollution when used with salt-tolerant plants such as Salicornia europaea. This statement improved the soil microenvironment, promoted a 43.9% increase in plant height, and enhanced soil microbial diversity [153].

### 4.3. Effects of Hydrogels on Plant Metabolism and Delivery of Beneficial Microorganisms

Hydrogels have excellent nutrient release properties, which can significantly enhance the efficiency of plant nutrient utilization and optimize primary metabolism [154,155]. For example, in a study on mustard, compared with directly using urea, controlled-release urea using galactomannan-chitosan hydrogels (HBWU) supplied nitrogen to plants more efficiently and stably. The chlorophyll content of plants treated with HBWU was significantly higher than that of the control group and the ordinary urea treatment group. Metabolomics analysis revealed that multiple key metabolites related to the nitrogen assimilation process were upregulated in the HBWU treatment group, effectively promoting the plant’s nitrogen metabolism pathway, thereby promoting growth and development [155].

**Figure 6:**
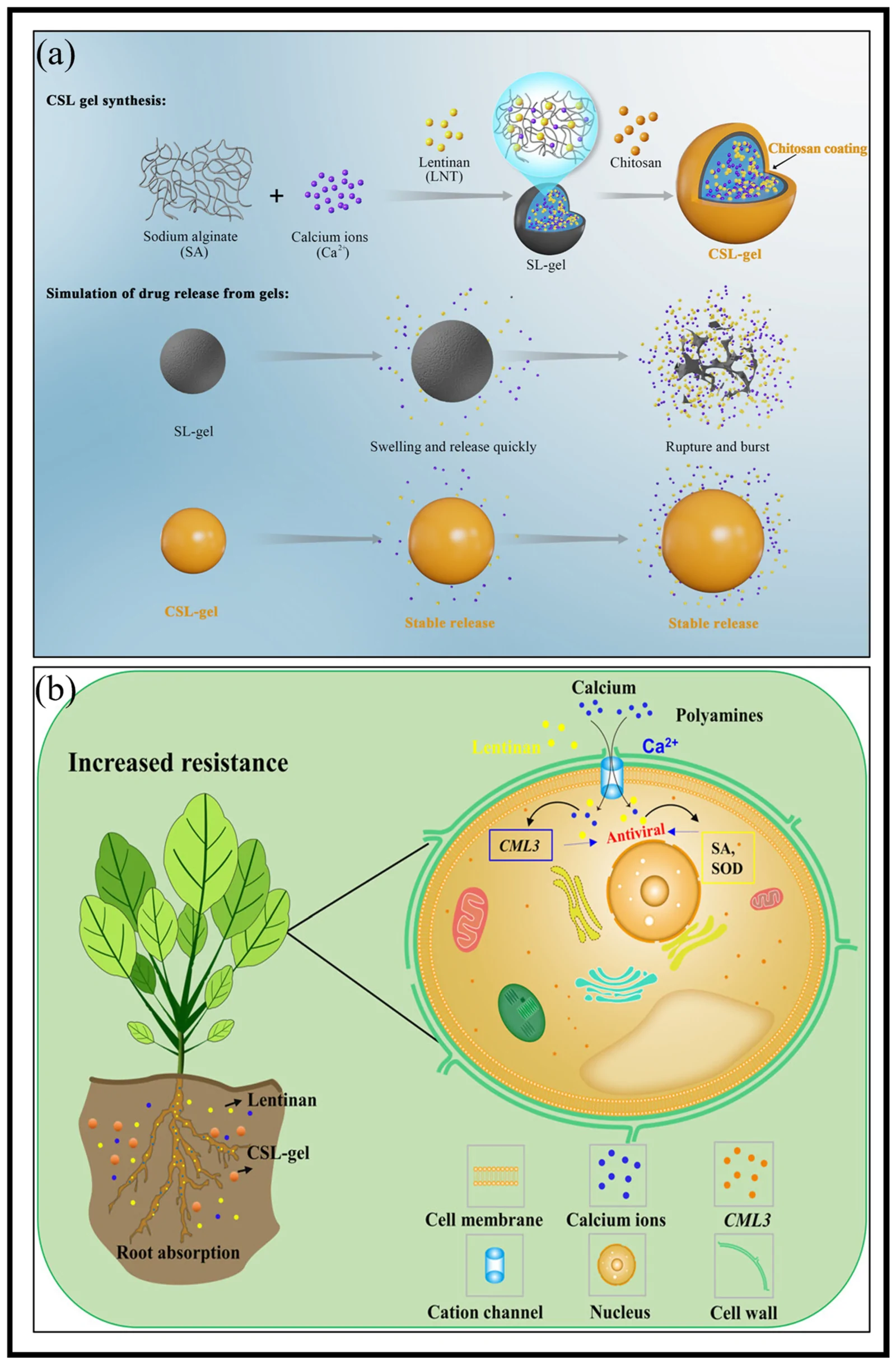
(**a**) Schematic diagram of CSL gel synthesis and drug release. (**b**) Schematic diagram of the proposed action model for the broad-spectrum resistance of different plant viruses triggered by CSL gel synthesized on the surface of the calcium alginate–lentinan (LNT) hydrogel (SL-gel. Reprinted with permission from reference [156]. Copyright 2023, Plant, Cell & Environment.

Another notable function of hydrogels is to enhance plant resistance to both biotic and abiotic stresses [156,157]. For instance, Southwest University developed a chitosan-hyaluronic acid calcium gel (CSL-gel) loaded with mushroom polysaccharides that continuously releases them and calcium ions. CSL-gel can stimulate systemic immunity in plants through a novel mechanism that regulates the calcium signaling pathway. This hydrogel treatment can significantly upregulate the expression of a calcium-binding protein called CML3 in Nicotiana tabacum. The CSL-gel induces broad-spectrum antiviral responses in plants by continuously releasing LNT (mushroom polysaccharides) and Ca^2+^ (calcium ions). LNT activates plant defense-related enzymes and the salicylic acid signaling pathway, while Ca^2+^ upregulates the expression of calcium-binding proteins, thereby enabling plants to have broad-spectrum resistance against various viruses such as tobacco mosaic virus and tobacco rattle virus (TMV and TRV). Thus, CSL-gels play a crucial role in promoting plant growth and providing long-lasting disease resistance through the dual pathways of immune activation and calcium signaling (Figure 6) [158].

**Figure 7:**
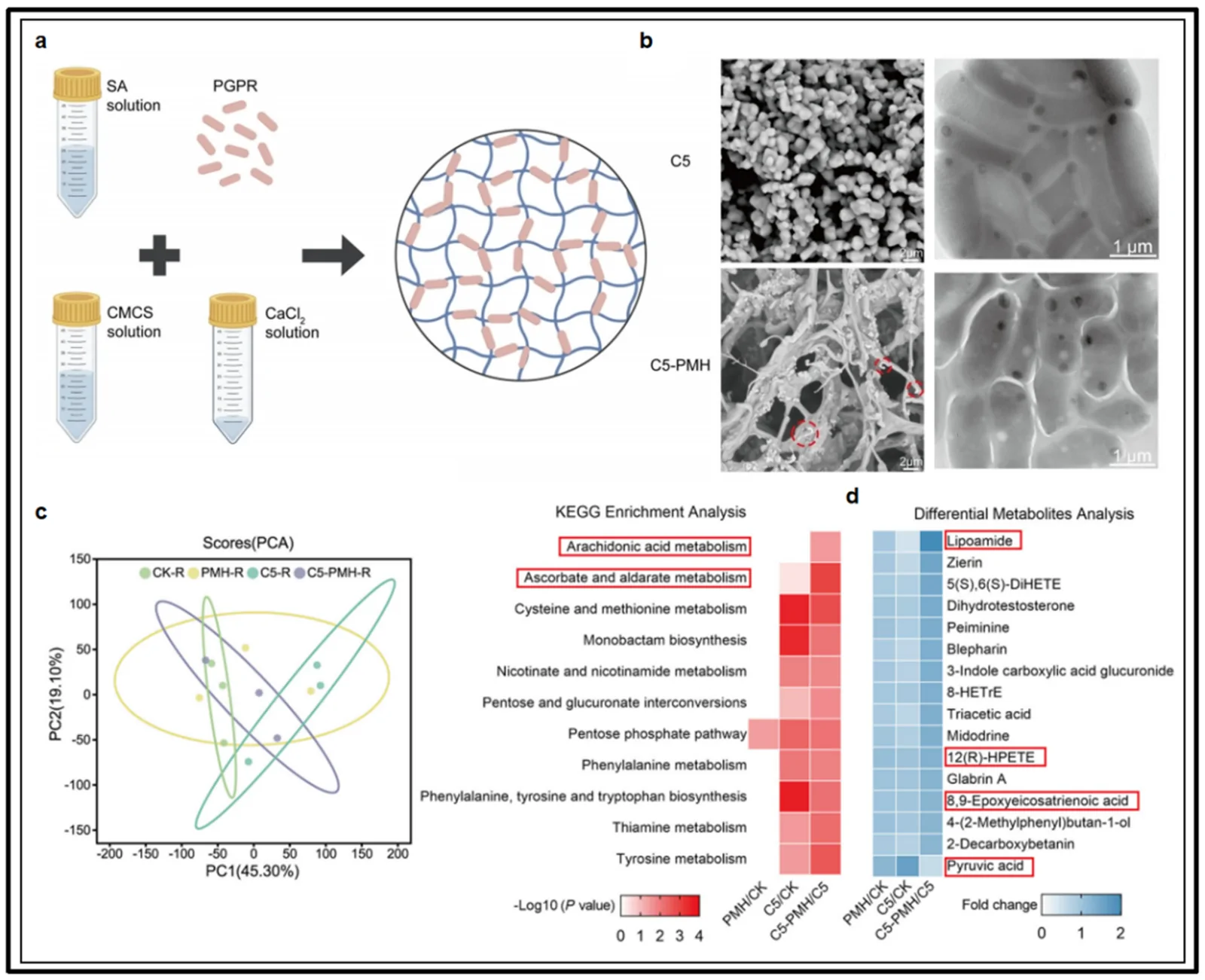
(**a**) Schematic diagram of the synthesis mechanism of polymeric hydrogel (PMH). (**b**) TEM images of C5 and C5-PMH, with red circles indicating the encapsulated C5 bacteria. C5-PMH shows that the bacteria are encapsulated by the hydrogel, while the free C5 has no outer structure. The scale is 1 micrometer. (**c**) Principal Component Analysis (PCA) of the root metabolites of cabbage-type rapeseed identified by LC-MS under the action of PMH. (**d**) Pathway analysis of the differentially enriched metabolites in the root of rapeseed (KEGG). Reproduced with permission from reference [141]. Copyright 2025, The Author(s) Qirui Feng et al.

Beyond water and nutrient retention, hydrogels can also alter plant growth status and stress resistance by regulating endogenous hormones and metabolic pathways. Additionally, they can indirectly modulate the plant metabolic state by influencing the rhizosphere microbial community [159]. For example, auxin is a core signaling molecule that regulates plant root and shoot development. Hydrogels can affect the balance and distribution of endogenous hormones, such as auxin, in plants. Studies have shown that polymer hydrogels made from carboxymethyl chitosan and sodium alginate (PMH) can carry beneficial rhizosphere bacteria. After encapsulating phytohormones with salicylic acid (SA), the mixture was combined with carboxymethyl chitosan and calcium chloride (CMCS and CaCl_2_) to prepare a biofertilizer (Figure 7a), which acts as a carrier to protect bacteria from the stress of acidic soil and promote their colonization in plant roots. At the same time, this hydrogel influences the microbial community structure in both the plant root and the surrounding rhizosphere soil. The surface and cross-section of PMH reveal a porous structure with interconnected pores, providing a high specific surface area that facilitates moisture retention and air permeability, thereby creating a suitable environment for bacterial reproduction. Scanning electron microscopy (SEM) revealed that the C5 was encapsulated in the spatial mesh structure of PMH, as delineated in Figure 7b.

To further investigate the mechanism of action of C5-loaded PMH (C5-PMH), they performed liquid chromatography mass spectrometry (LC-MS) untargeted metabolomics analysis on roots of oilseed rape samples. Principal component analysis (PCA) showed that wrapping C5 with PMH significantly altered the metabolites of oilseed rape roots, as shown in Figure 7c. Furthermore, the annotated biological processes of metabolites were summarized by Kyoto Encyclopedia of Gene and Genomes (KEGG) enrichment analysis; the “arachidonic acid metabolism” pathway and the “ascorbate and aldarate metabolism” pathway were significantly enriched in the C5-PMH group (Figure 7d). The variable importance in projection (VIP) score was utilized to determine the separation potential of the metabolites. According to our predefined criteria (p1 and |fold change| > 1.2), 16 metabolites were demonstrated to be differentially expressed after wrapping C5 with PMH (Figure 7c). Conversely, it was found that arachidonic acid metabolism and the ascorbate and aldarate metabolism pathway were significantly upregulated under C5-PMH, while pyruvic acid and lipoamide pathways were significantly downregulated. This helps enhance plant defense responses and improve plant growth efficiency, while the decrease in Pyruvic acid suggests that sugar metabolism shifts towards energy production rather than accumulation, thereby optimizing energy utilization.

The microbial community composition of the roots and rhizosphere soil of rapeseed treated with PMH and specific strains shows significant differences from that of the control group. These successfully colonized bacteria regulate the microenvironment of the root, influence the deposition of suberin-like substances in the endodermis, and guide auxin accumulation at the root tip. This directly promotes the initiation and elongation of lateral roots, enhancing the plant’s ability to absorb water and nutrients [141].

## 5. Strategies for the Application of Hydrogels at Different Plant Growth Stages

### 5.1. Seeds and Germination Stage

**Figure 8:**
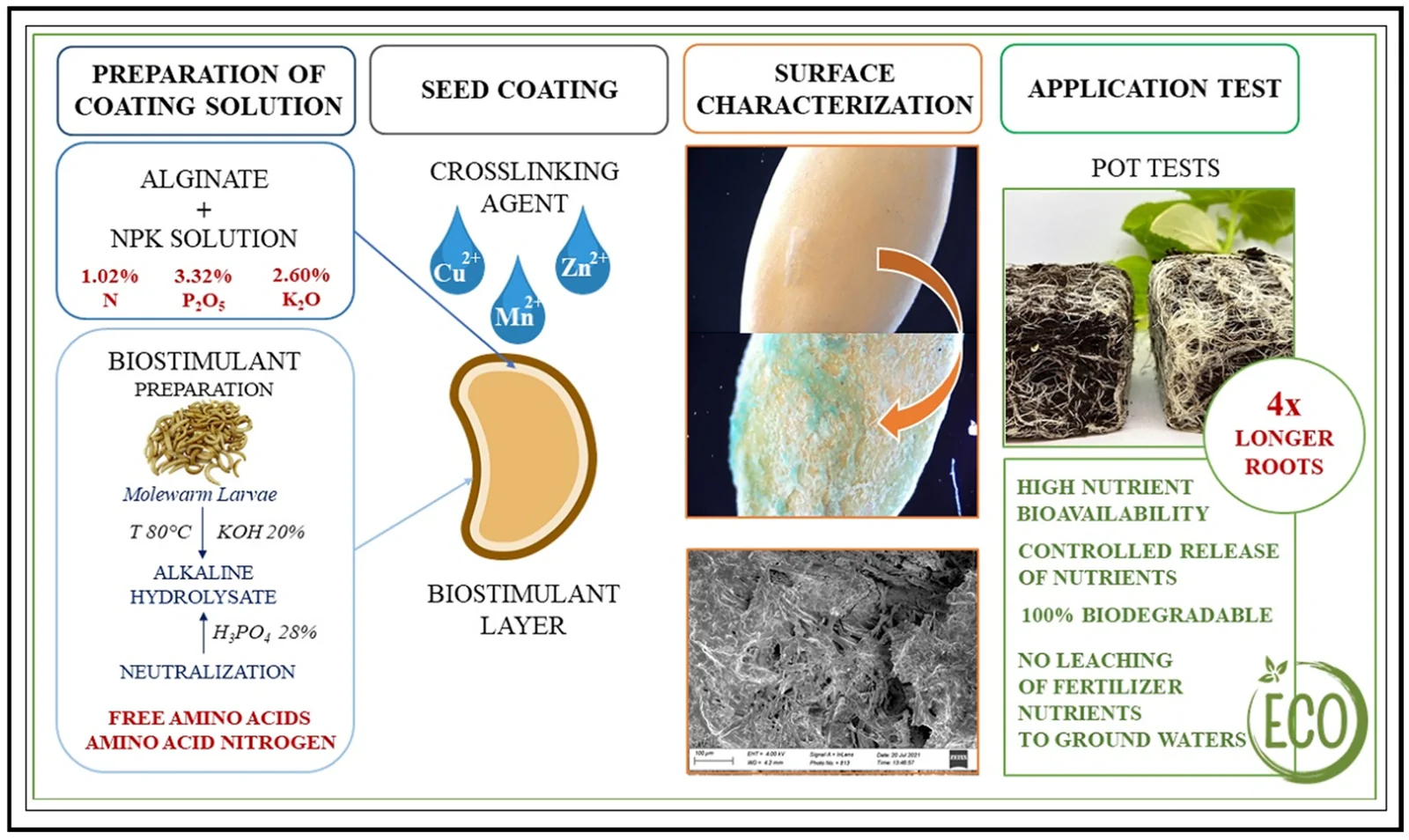
Alginate aqueous gel seed coating. Reproduced with permission from reference [160].

During the seed germination stage, hydrogels can be applied for tasks such as seed coating [143]. Due to their three-dimensional network structure and hydrophilic groups, hydrogels can absorb hundreds of times their own weight in water, forming a localized water storage environment around the seeds [143,160]. When environmental moisture is sufficient, hydrogels store water; when conditions become dry, they slowly release water, providing a continuous and stable water supply. This creates a stable, moist microenvironment for seed germination, thereby significantly increasing the germination rate and uniformity of seed emergence [9]. For example, when gellan hydrogel is used in wheat seed coating, coated seeds exhibit a higher germination rate and greater biomass accumulation, and their survival time under water-deficient conditions is 10 days longer than the control group [161]. As a functionalized platform, hydrogels can also carry various functional components, endowing seeds with enhanced tolerance to adverse conditions such as salinity and certain diseases. For instance, in Figure 8, the researchers used a 2% sodium alginate (SA) solution as the base material and applied the alginate solution containing NPK to the surface of the seeds by immersion or spraying. They then cross-linked and solidified it into a hydrogel seed coating with a Cu^2+^/Zn^2+^/Mn^2+^ micronutrient solution, and coated the outer layer with the alkaline hydrolyzed amino acid solution of the mealworm to act as a biological stimulant. In terms of the effect, the nutrient leaching in the water was ≤10%, and the bioavailability of NPK and trace elements reached 60–100%, proving that the nutrients could be effectively absorbed in the root zone. Compared with uncoated seeds, the fresh weight of cucumber seedlings increased by approximately 50%, and the root length increased by approximately four times, promoting early growth and being biodegradable [162]. Researchers from the Chinese Academy of Agricultural Sciences (CAAS) encapsulated Bacillus subtilis ZF71 in a sodium alginate-polyacrylamide hydrogel and used it for cucumber seed coating. The SA/PC hydrogel loaded with ZF71 was coated onto the surface of cucumber seeds using an immersion coating method. Ultimately, the SA/PC hydrogel loaded with ZF71 formed a film, and ZF71 cells were observed to aggregate into a layer on the seed surface. This hydrogel can form a biomimetic membrane-like structure on the seed surface, maintaining a high viable bacterial count on the seeds after 37 days of storage. After sowing, the hydrogel helps beneficial bacteria effectively colonize the root system of cucumbers, achieving a 53.26% control effect against Fusarium root rot, which is significantly superior to direct application of bacterial suspension [143].

### 5.2. Nutritional Growth Period

During the vegetative growth period of plants, stems, leaves, shoots, and roots develop rapidly, and the plant has a high and sensitive demand for water and nutrients. The application of hydrogels at this stage should focus on regulating the release rhythm of water and nutrients to match the crop’s nutrient requirement patterns, thereby improving water and nutrient use efficiency [163,164]. At the same time, hydrogels can also be used to improve soil structure and regulate rhizosphere microecology, maintaining continuous moisture and air permeability in the root zone, preventing drought or waterlogging stress, optimizing the rhizosphere microenvironment, protecting and promoting plant root growth, and enhancing plant stress resistance and nutrient absorption efficiency. For example, in a water-deficient sodium alginate-G-polyacrylamide hydrogel under water-deficient conditions, the survival rate of eucalyptus plants significantly increased. The sodium alginate-g-polyacrylamide (4Alg:1PAM) hydrogel promotes plant development during the vegetative stage through a synergistic triad of moisture-retentive, slow-release properties, rhizospheric nutrient regulation, and physiological activation. Exhibiting an equilibrium swelling capacity of 65 g/g, the matrix retains over 30% of its stored water after five days at 30 °C and 30% relative humidity, thereby absorbing irrigation inputs and gradually desorbing moisture under deficit conditions to mitigate vegetative-phase water stress; consequently, the relative root-collar diameter growth rate of water-deficient Eucalyptus seedlings reached 0.012 mm·d^−1^, approximating 80% of that recorded under full irrigation. Soil incubation trials revealed an ~85% degradation rate within five weeks, wherein hydrolytic cleavage of alginate glycosidic bonds yields oligosaccharides that function as elicitors/biostimulants to enhance drought acclimation. Concurrently, the polyanionic character of alginate elevates soil cation exchange capacity, improving NH_4_^+^ retention and facilitating sustained Ca^2+^ release to meet peak vegetative mineral demands. At the physiological level, hydrogel application stabilized predawn leaf water potentials between −1.43 and −1.72 MPa, elevated net photosynthetic rates to 3.11–3.51 μmol·m^−2^·s^−1^, and increased instantaneous water-use efficiency to 20.66–23.44 μmol·mol^−1^, collectively sustaining carbon assimilation and aboveground biomass accrual. Treated seedlings also exhibited greater relative height increments and an increased leaf-to-stem ratio, preserving functional foliage and markedly reducing sapling mortality in arid environments—thus demonstrating strong agronomic efficacy in promoting vegetative growth [67].

### 5.3. Reproductive Growth Period

During the reproductive growth period of plants, hydrogels can support high-yield, high-quality crop production through intelligent water and fertilizer management, enhancing stress resistance and optimizing the root zone environment. They supply water and nutrients during the fruit expansion and ripening stages, improve fruit quality, and can also be used for fruit monitoring and preservation [165,166]. For instance, the hybrid hydrogel (GAXH) constructed through intermolecular interactions between flaxseed arabinoxylan and Acacia senegal gellan gum significantly improved the water retention capacity of sandy soil in the cherry tomato pot experiment. At 35 °C, it retained 140% of soil moisture for 30 days. Cherry tomatoes grown in GAXH-treated soil had significantly higher levels of lycopene, total carotenoids, and total polyphenols in their fruits compared with the control group, and their antioxidant activity was also enhanced [167].

In the postharvest stage, hydrogels demonstrate dual capacities for intelligent monitoring and active preservation. The hydrogel-based intelligent packaging system can visually monitor fruit freshness by integrating antibacterial, antioxidant, and sensing functions. For instance, the CuLyz nanozyme hydrogel film (PVA/TA/CuLyz) is utilized for post-harvest preservation of strawberries. Operating via a multi-tiered mechanism, tannic acid crosslinks polyvinyl alcohol into a robust hydrogen-bonded network that uniformly immobilizes the self-assembled Cu^2+^-lysozyme nanozymes, thereby conferring a tensile strength of 3.49 MPa and an elongation at break of 615% to effectively absorb transit-induced mechanical shocks. Functionally, the phenolic hydroxyls of tannic acid and the nanozyme exert synergistic bioactivity, achieving an 83.5% DPPH radical-scavenging rate and 96% and 95% inhibition against Escherichia coli and Staphylococcus aureus, respectively. The resultant dense architecture concurrently provides UV shielding and restricts moisture/oxygen transmission, reducing strawberry weight loss to merely 10.3% over six days—markedly lower than the 31.2% in untreated controls—and delaying both firmness deterioration and organic acid depletion. Leveraging its intrinsic strain-sensing capability and the YOLOv8 machine learning algorithm, the film enables real-time detection of concealed bruising and incipient decay. Under ambient conditions of 23–26 °C, this system extends strawberry shelf life by 6 days, with the increase in total viable count curtailed to 42% of the control baseline. Collectively, hydrogel technologies now span the entire agricultural value chain—from field-level fruit development and quality formation to intelligent postharvest preservation—offering a unified materials science paradigm to stabilize yields, improve quality, and minimize harvest losses in high-value crops.

## 6. Economic Benefits and Cost Considerations of Hydrogels

The economic benefits of hydrogels are reflected in multiple aspects, including water conservation, increased crop yield, and reduced fertilizer usage [168]. For instance, amending soil with 2% of carboxymethyl cellulose sodium (NaCMC) and hydroxyethyl cellulose (HEC) can increase water-use efficiency by 25.25% to 45.52% in planted crops. The stable water supply creates a superior environment for crop growth, thereby increasing both yield and the quality of agricultural products [68]. At the same time, hydrogels can serve as carriers for fertilizers and pesticides, achieving sustained release effects while simultaneously reducing fertilizer usage and pollution. This significantly improves the utilization rate of fertilizers and pesticides, reduces their application rates, and minimizes environmental pollution [134]. For example, a stretchable double-network nano-composite hydrogel pesticide sustained-release delivery system was prepared using sodium alginate (SA), polyacrylamide (PAM), and montmorillonite (MMT) [169].

Hydrogels also bring important indirect and long-term benefits. For instance, by reducing the frequency of irrigation, hydrogels save related labor costs [7]. They also help enhance crop drought resistance under fluctuating water conditions. Biodegradable hydrogels, particularly those based on natural polymers such as cellulose, can decompose in the environment, thereby avoiding long-term soil pollution and meeting the requirements of green agriculture. Hydrogels also offer various environmental benefits that contribute to the sustainability of agricultural practices. For example, hydrogels can help slow down soil erosion by stabilizing soil structure and reducing surface runoff. In sloped or degraded land, the use of hydrogels has been shown to improve soil retention and prevent the loss of valuable topsoil and nutrients. Additionally, hydrogels can assist in soil remediation by adsorbing and immobilizing certain pollutants [170].

Although hydrogels have many advantages in agriculture, their high cost still limits their development and application in this field. In particular, high-quality hydrogels extracted from synthetic polymers have higher production costs and more complex manufacturing processes compared with traditional fertilizers, making it difficult to reduce costs in a short period. As a result, these hydrogels remain inaccessible to ordinary small-scale farmers [171]. Although the long-term benefits of hydrogels, such as water conservation, increased yield, reduced fertilizer usage, and lower environmental risks, are significant, the initial investment required for hydrogels and the lack of obvious short-term economic returns may not be appealing to the average farmers [147]. Moreover, because the economic benefits can be uncertain or depend on factors such as crop types, soil conditions, and local climate factors, the water-saving and yield increase effects of hydrogels may not be consistently observed.

The total cost of hydrogel application mainly includes the cost of the materials themselves and the associated application costs. The raw materials and synthesis process of traditional hydrogels are the primary cost drivers. However, through material innovation, this cost can be significantly reduced. For example, using the full components of agricultural waste to prepare hydrogels enables resource recycling and lowers raw material costs [172]. Additionally, combining hydrogels with other technologies can reduce the need for extra labor or equipment investment, thereby lowering the technical threshold and application costs. For example, a hydrogel-reverse osmosis (RO) integrated system, used as a pre-treatment method for reverse osmosis processes, can significantly reduce the operating pressure and energy consumption in the RO stage, achieving an overall energy-saving rate of 51%. Compared with conventional RO systems, this approach also results in substantial reductions in membrane component investment and maintenance costs [173]. Future development of hydrogels should focus on material innovation and optimization models to further improve their cost-effectiveness ratio and enable the technology to play a more significant role in sustainable agriculture. In addition, government subsidies, fiscal incentives, or cost-sharing programs can be adopted to alleviate farmers’ economic burden and encourage the adoption of hydrogel technology.

## 7. Current Challenges and Environmental Issues of Hydrogels

In the field of agriculture, although hydrogel technology has a promising future and has been applied in many aspects, hydrogels still face multiple challenges in large-scale applications. These include limitations related to the material itself, environmental factors, and ecological safety [174].

The application of hydrogels is accompanied by challenges related to biodegradability and environmental residue risks [175]. For example, traditional synthetic hydrogels that use polyacrylamide (PAM) as the matrix, commonly used in agriculture, are often not effectively decomposed by microorganisms in natural environments [176]. After their period of use, these materials may remain in the soil for a long time, forming organic microplastics that are difficult to remove and causing secondary pollution. What is more concerning is that residual monomers, such as acrylamide, remaining from the chemical synthesis process have been shown to be toxic to soil microbial communities, thereby disrupting the biological foundation of soil health [177].

Hydrogels must withstand various mechanical stresses and complex environmental conditions in practical applications. Their mechanical properties and long-term stability are the current research focus and challenges [178]. Many traditional hydrogel materials are typically soft and fragile, with tensile strength often lower than 1 megapascal (MPa) and limited elongation at break, making them unable to meet the requirements of applications that need to withstand large or repeated deformations [179]. Although strategies such as constructing a double-network structure can be used to improve hydrogel performance, achieving a balance between strength and toughness while also incorporating self-healing ability remains a significant challenge [180]. Hydrogels are prone to failure in extreme environments [181]. For example, in high-salt environments, high concentrations of chloride ions can penetrate the hydrogel network, triggering ion exchange and changes in osmotic pressure. This may lead to excessive swelling of the hydrogel, a sharp decline in mechanical properties, and even structural disintegration [181,182]. Agricultural applications typically require hydrogels to maintain structural integrity under dry–wet cycles and soil mechanical pressures [183]. However, many hydrogels, especially those with high water absorption, often have poor mechanical strength. Under multiple absorption-release cycles or tillage pressure, their three-dimensional network structure is prone to damage, leading to functional degradation and reducing long-term water retention. Developing hydrogels with self-healing properties is a strategy to improve their durability [184]. For example, by combining dynamic borate ester bonds and Hofmeister effects, repair can be achieved through a two-step “salt fusion-salt condensation” method, with repair efficiency approaching 100% within 95 min and strength reaching up to 23 MPa after repair. The repair method enables rapid repair under high strength and allows the material to maintain high-intensity impact and wear resistance, significantly extending its service life under harsh conditions [185].

The biocompatibility of hydrogels sometimes conflicts with the requirements for achieving specific functions [186]. For example, using biocompatible poor synthetic monomers or toxic crosslinking agents can impart excellent mechanical properties or specific functions to hydrogels; however, the synthesis and use of hydrogels may leave harmful substances that cause adverse reactions [187]. Even with natural polymers, their crosslinking process or modification treatment may introduce potential irritants or toxic substances [188]. The process of modifying hydrogels for specific functionalization, such as pH response, temperature response, light response, conductivity, or antibacterial properties, is often complex and typically requires the introduction of additional chemical components or nanomaterials into the network [189]. However, these additives may alter the physicochemical properties of the hydrogel and may also bring about new biosafety concerns, which require systematic and rigorous assessment.

Although hydrogels can improve soil structure [190], the non-target effects that long-term, large-scale application of hydrogels and their degradation process may have on the soil ecosystem remain unclear. It is still unknown whether the prolonged presence of hydrogels will have unexpected impacts on the formation of native soil aggregates, pore distribution, air permeability, soil organic matter cycling, and the carbon–nitrogen balance [191]. Furthermore, whether hydrogels, as exogenous additives, have negative effects on microbial community diversity and activity, the safety of soil fauna, or the microecology of the rhizosphere after being added to the soil remains to be determined [192]. The complex interactions between hydrogels and the environment currently lack sufficient long-term data to provide an explanation.

## 8. Conclusions and Future Prospects

### 8.1. Main Findings

**Figure 9:**
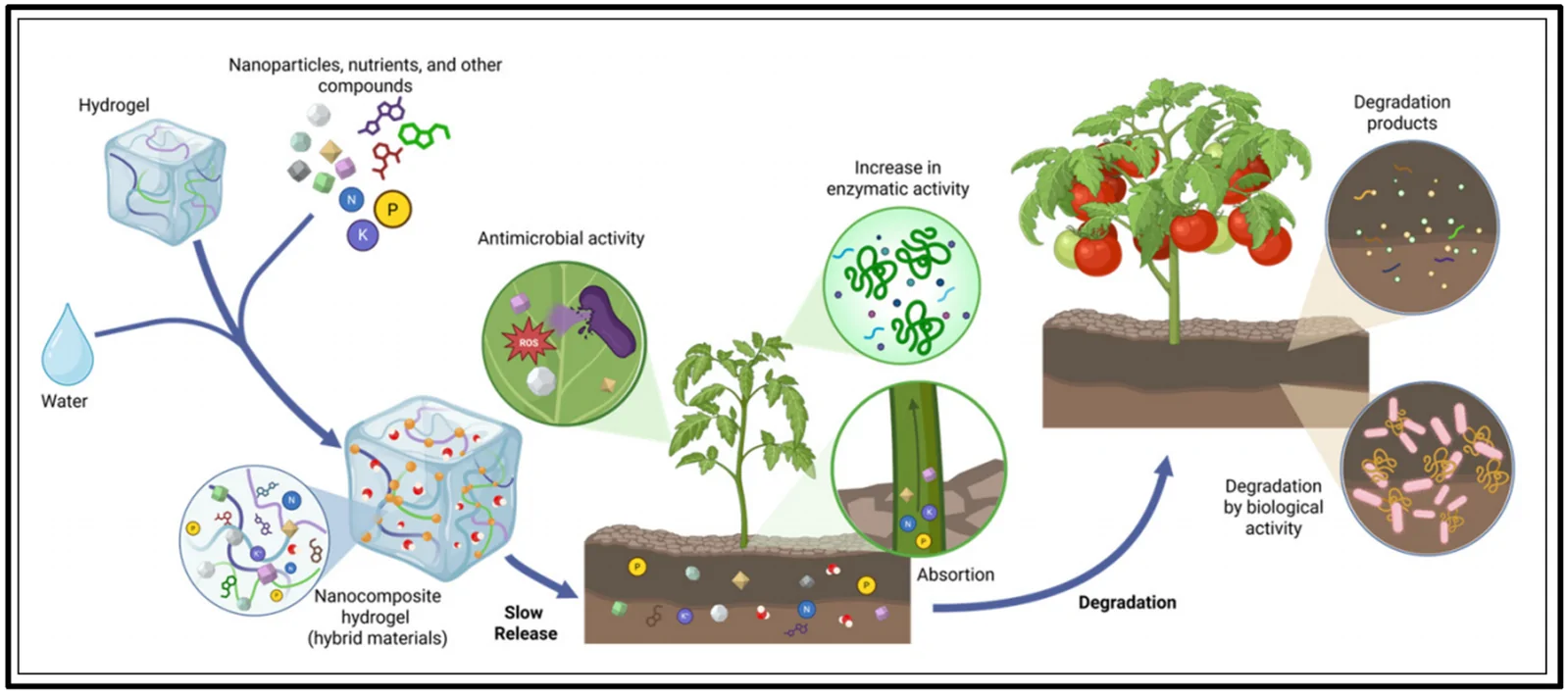
Schematic diagram illustrating the dual action mechanism of nanocomposite hydrogels in crops. At the soil–root interface (left image), the polymer hydrogel matrix acts as a micro-storage reservoir, capable of absorbing and storing water and dissolved nutrients, reducing water loss through osmosis and evaporation, and releasing agricultural chemicals in a controllable manner, thereby improving water-use efficiency and fertilizer utilization. At the plant level (right image), the encapsulated nanoparticles, plant chemicals, and biological stimulants act as physiological stressors, triggering antioxidant defense, hormone balance regulation, and accumulation of bioactive metabolites. The synergistic effect of these physical and physiological processes can significantly enhance seed germination rate, growth speed, yield stability, and nutritional quality under both abiotic and biotic stress conditions. Reproduced with permission from reference [58].

Hydrogels, as an advanced functional material, are playing an increasingly important role in agriculture. Their unique three-dimensional hydrophilic network structure and controllable physicochemical properties give them great potential for addressing agricultural challenges, including water scarcity, low fertilizer utilization rates, lack of field-scale validation, and environmental pollution [193]. Owing to their exceptional water retention and sustained-release capabilities, as well as their adsorption capacity for heavy metal ions, hydrogels can effectively improve soil structure, stabilize moisture and nutrient regimes in the root zone, and sequester heavy metals from the soil. Consequently, this mitigates the phytotoxic effects of heavy metals while enhancing crop water-use efficiency and nutrient uptake [15]. Hydrogel is rapidly emerging as a green, ecofriendly, and climate-smart strategy for enhancing crop drought resistance. It triggers defensive crop physiological, anatomical, as well as molecular reprogramming during high yield, thereby enhancing crop growth performance under subsequent abiotic stress conditions (such as drought and heavy metals). Figure 9 illustrates the mechanism of action of the hydrogel in crops [58].

Hydrogels can also promote the development of plant root systems and enhance the resistance of crops to abiotic stresses such as drought, heavy metals, and salinity [148]. Through functional design, hydrogels have also played important roles in heavy metal adsorption, pesticide residue degradation, and as carriers for beneficial microorganisms. However, the large-scale application of hydrogels in agriculture still faces a series of challenges [194]. These obstacles include the poor biodegradability and potential environmental persistence of conventional synthetic hydrogels, inadequate mechanical strength coupled with compromised long-term stability under complex field conditions, and the persistent difficulty of balancing performance and cost in hydrogels derived from natural polymers. Further concerns regarding the long-term, non-target effects of hydrogels on soil ecosystems, combined with high initial investment costs, collectively constrain their widespread adoption among farmers.

### 8.2. Future Prospects

Although current agricultural hydrogels have demonstrated notable potential for water retention and yield improvement at the laboratory scale, realizing their widespread adoption requires shifting the research paradigm from proof-of-concept to metric-constrained engineering R&D. First, region-specific standardized testing protocols and market-access criteria must be established. Beyond conventional water absorption capacity, mandatory core metrics should include a water-retention rate ≥ 60% after ≥3 field wet–dry cycles and an in situ soil biodegradation rate ≥ 70% within 180 days. Differentiated performance baselines are needed for distinct soil textures—for instance, sandy-soil formulations must increase plant-available water by ≥40% at 45% field capacity, while clay-targeted hydrogels must not reduce soil aeration porosity by more than 5%.

Beyond material advancement, commercial scalability and cost reduction remain pivotal to accessibility for smallholders. The dry-mass production cost of biomass-derived multifunctional hydrogels should be driven below the break-even threshold of international commodity-grade agricultural superabsorbent polymers (SAPs). This demands prioritizing one-pot graft polymerization of agricultural wastes—such as straw and pomace—and bypassing intensive purification steps to reduce processing energy consumption by over 30%.

Future applications should decode hydrogel–soil interactions to develop soil-tailored hydrogels that couple material innovation with environmental safety [195]. Threshold-responsive intelligent architectures should be deliberately designed; for example, triggering automatic water release at a soil matric potential ≤−30 kPa or initiating nutrient release when rhizosphere pH drops below 6.0, thereby transitioning hydrogels from passive water retainers to smart rhizosphere micro-environment regulators. Such systems can be fabricated from agrowaste to achieve low-cost, fully biodegradable, and on-demand co-delivery of water, fertilizers, and agrochemicals [172].

The hydrogel should be more deeply integrated into the precision agriculture infrastructure to expand its application scope from staple grains to high-value protective crops and marginal lands (such as saline–alkali lands and desertified areas) [196]. On the policy front, with reference to the rollout model for bio-organic fertilizers, tiered subsidy pilots could be introduced in key production zones to incorporate hydrogel application into high-standard farmland development quotas. Crucially, multi-year (≥3 yr) stationary field trials across ecoregions are urgently required to map soil microbiome succession and carbon–nitrogen footprints, systematically evaluate long-term ecological impacts, and codify science-based safety assessment industry standards.

## Data Availability

No new data were created or analyzed in this study. Data sharing is not applicable to this article.
